# Biclustering analysis on tree-shaped time-series single cell gene expression data of *Caenorhabditis elegans*

**DOI:** 10.1186/s12859-024-05800-y

**Published:** 2024-05-09

**Authors:** Qi Guan, Xianzhong Yan, Yida Wu, Da Zhou, Jie Hu

**Affiliations:** School of Mathematical Sciences, Xiamen University, Xiamen, 361005 Fujian China

**Keywords:** Single-cell gene expression, Tree-shaped dataset, Biclustering, Genetic algorithm

## Abstract

**Background:**

In recent years, gene clustering analysis has become a widely used tool for studying gene functions, efficiently categorizing genes with similar expression patterns to aid in identifying gene functions. *Caenorhabditis elegans* is commonly used in embryonic research due to its consistent cell lineage from fertilized egg to adulthood. Biologists use 4D confocal imaging to observe gene expression dynamics at the single-cell level. However, on one hand, the observed tree-shaped time-series datasets have characteristics such as non-pairwise data points between different individuals. On the other hand, the influence of cell type heterogeneity should also be considered during clustering, aiming to obtain more biologically significant clustering results.

**Results:**

A biclustering model is proposed for tree-shaped single-cell gene expression data of *Caenorhabditis elegans*. Detailedly, a tree-shaped piecewise polynomial function is first employed to fit non-pairwise gene expression time series data. Then, four factors are considered in the objective function, including Pearson correlation coefficients capturing gene correlations, *p*-values from the Kolmogorov-Smirnov test measuring the similarity between cells, as well as gene expression size and bicluster overlapping size. After that, Genetic Algorithm is utilized to optimize the function.

**Conclusion:**

The results on the small-scale dataset analysis validate the feasibility and effectiveness of our model and are superior to existing classical biclustering models. Besides, gene enrichment analysis is employed to assess the results on the complete real dataset analysis, confirming that the discovered biclustering results hold significant biological relevance.

**Supplementary Information:**

The online version contains supplementary material available at 10.1186/s12859-024-05800-y.

## Introduction

**Fig. 1:**
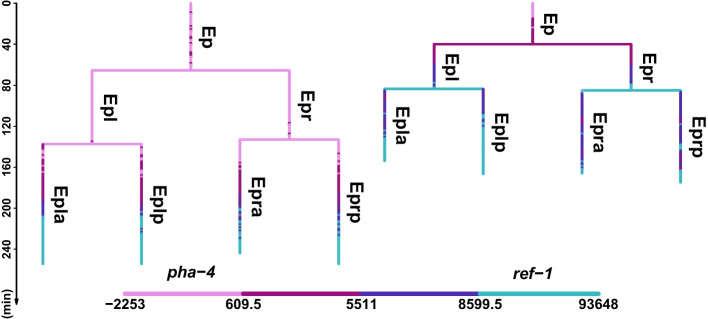
An example of real data. The figure illustrates cell lineage subtrees from two genes, pha-4 and ref-1, where each horizontal line represents a cell division event, and each vertical line represents a cell. The cell names are annotated on the right side of each line. The length of the vertical line is proportional to the cell’s lifetime, and the color of the lines corresponds to the fluorescence intensity of the labeled genes

The process of how a single-cell fertilized egg develops into adulthood is a fundamental yet unsolved problem in biology, where gene-selective expression plays a crucial role [1]. Due to the transparency and consistent cell lineage, *Caenorhabditis elegans* (*C.elegans*) has remained a vital model organism in molecular biology and developmental biology [2]. Especially, with the emergence of time-lapse confocal laser microscopy technology developed by [3, 4], researchers can conduct further analysis to quantitatively examine the expression patterns of various genes and their relationships with cell fates [5–7]. The real dataset produced by such technology traces the time-series fluorescence intensity of labeled genes within each cell. Starting from the organism’s developmental origin, each cell divides into two new cells, thus forming a binary structure. By tracking its developmental process, such binary tree-shaped time-series data is generated. Each data file records the expression of one labeled gene on one *C.elegans* individual and can be considered as tree-shaped single-cell gene expression data. Figure 1 displays examples of cell lineage subtrees from two data files. Each horizontal line represents a cell division event, and the length of each vertical line corresponds to the lifetime of a single cell. Compared to scRNAseq data, tree-shaped data clearly displays the cell lineage relationship, eliminating the need for inferring pseudotime. Therefore, tree-shaped data enables researchers to more easily track and understand the dynamic changes in gene expression during the development and differentiation processes of organisms. Although such dataset provides dynamic gene expression information within single cells, the gene expression patterns at cellular level have not been well understood. Interested readers can refer to [8] for a probabilistic conception regarding tree-shaped datasets.

Gene clustering analysis is a method used to explore genes functions, aiming to group genes based on their expression patterns under different experimental conditions. The goal of traditional clustering algorithms is to identify non-overlapping sets of genes that exhibit similar expression patterns across all experimental conditions, typically partitioning the data solely based on a single dimension [9–12]. In contrast to traditional clustering, biclustering can capture similar gene expression patterns in specific subsets of conditions (such as specific cells), revealing critical genetic pathways [13, 14]. Cheng and Church [15] first applied biclustering to gene expression data, leading to the emergence of more effective biclustering algorithms [16–22]. These algorithms have played an important role in understanding various aspects of gene regulation, evolution, development, and disease mechanisms.

Despite the great promise of biclustering methods, effectively capturing analogous gene expression patterns within distinct conditions on tree-shaped single cell gene expression data involves two key challenges. First, while *C.elegans* shares consistent cell lineages, the lifetimes of the same cell in different embryos are usually different. Thus, since the measurement intervals are all 1.5 min in each experiment, non-pairwise time-series data points are recorded for different target genes. Taking Fig. 1 as an example, the two subtrees both start from the ‘Ep’ cell at time zero, but the data points within two subtrees are non-pairwise. Therefore, it is hard to computing correlations between genes based on the raw data. Second, with the availability of cellular and temporal information, it is crucial to consider the parent–child relationships during cell division within each subtree and the correlation between temporally adjacent data points within each cell. However, conventional biclustering algorithms primarily cater to gene-cell (or gene-tissue) count data and lack the ability to tackle time-series data.

These challenges are overcome by proposing a Tree-Shaped single-cell gene expression data Biclustering model for *C.elegans*. The model initially utilizes piecewise polynomial functions to fit the tree-shaped gene expression data. Subsequently, by considering the entire gene expression data, an objective function for the biclustering model is introduced and solved using Genetic Algorithm (GA). Finally, experiments using both small-scale and complete real datasets are completed.

## Materials and methods

**Fig. 2:**
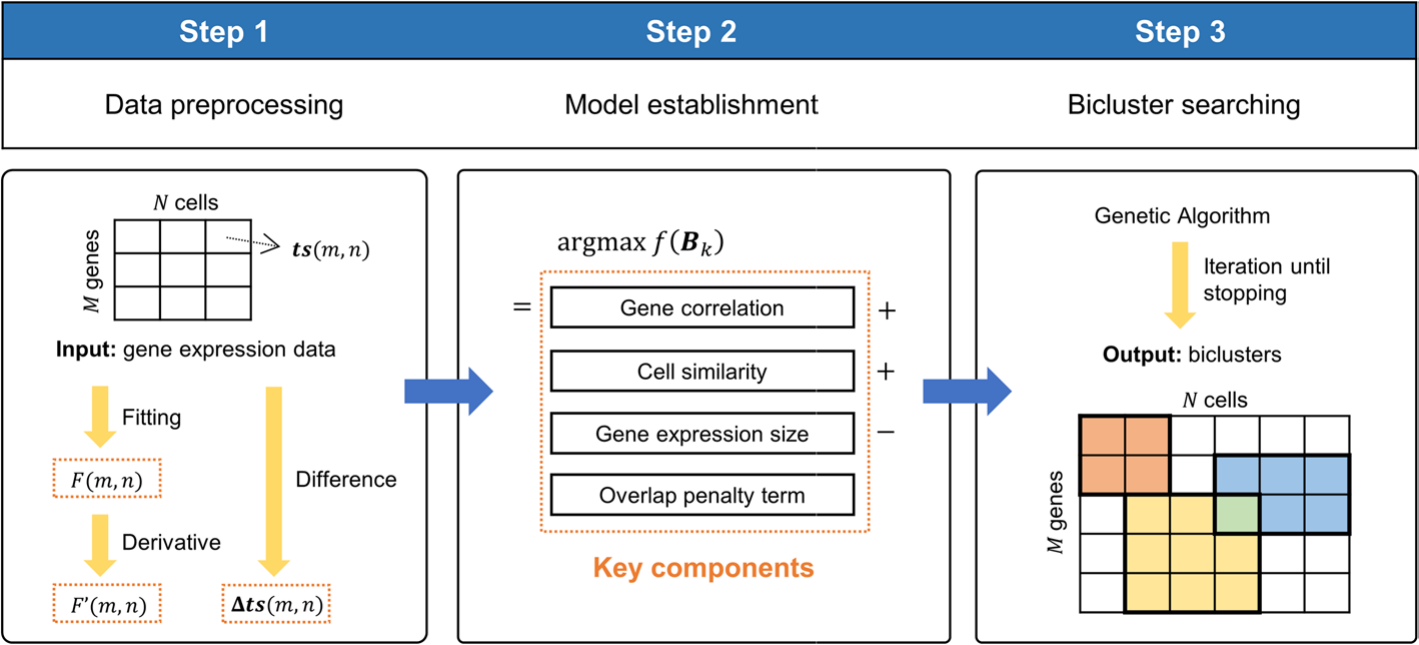
TSBic overview. Here, $\varvec{ts}(m, n)$ represents gene expression data, *F*(*m*, *n*) represents piecewise polynomial functions, $F^{\prime }(m, n)$ representing gene expression rate functions, and $\varvec{\Delta ts}(m, n)$ represents gene expression rate data

A $\textbf{T}$ree-$\textbf{S}$haped single-cell gene expression data $\textbf{Bic}$lustering model for *C.elegans* is proposed (TSBic). The overview of TSBic is shown in Fig. 2, and the TSBic method consists of the following three-step approach:

$\bullet$ *Step 1*: Preprocessing data for subsequent analysis.

$\bullet$ *Step 2*: Establishing the objective function and setting hyper-parameters of the biclustering model.

$\bullet$* Step 3*: Applying GA to search for biclusters until the stopping criterion is satisfied.

All experiments in this study are conducted on a server equipped with 4 CPUs (Intel (R) Xeon (R) Platinum 8270 with 2 threads * 26 cores, @ 2.70GHz), 6.5TB of non-system disk storage, and 1TB of RAM. The code uses R 4.1.3 as the primary programming language.

### Data preprocessing

The real dataset used in this experiment can be found at http://epic.gs.washington.edu/ [5]. Due to limited observation time, missing values are calculated for each cell and each gene. Genes and cells with missing value proportions exceeding 60% are subsequently removed.

After then, let $\varvec{X}_{M \times N}$ denote the gene expression data matrix, where rows correspond to genes, and columns represent cells. Each entry of the matrix are time series data $\varvec{ts}(m, n)$, indicating the expression data of the *m*-th gene within the *n*-th cell, and remaining missing data in $\varvec{X}$ are not involved in the subsequent calculation. Besides, based on the gene expression onset detection by [6], a binary matrix $\varvec{Y}_{M \times N}$ indicating the gene expression 0-1 matrix is constructed, where $y_{mn}=1$ signifies that gene *m* was expressed in cell *n*, while $y_{mn}=0$ indicates that gene *m* was not expressed in cell *n*.

**Fig. 3:**
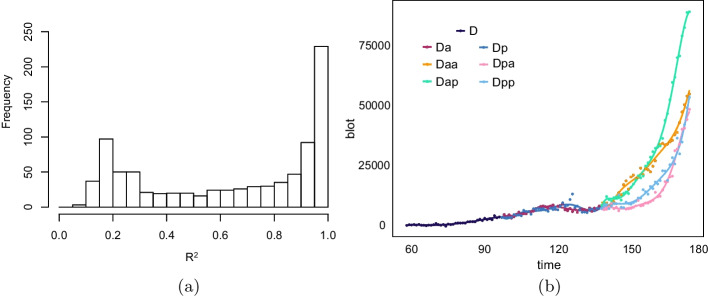
Fitting result. **a** Histogram of coefficient of determination $R^2$. The mean value of $R^2$ is 0.64, the standard deviation is 0.32. The 25% percentile is 0.3, the 50% percentile is 0.75, the 75% percentile is 0.95, and the 100% percentile is 0.98. **b** Time series plot of gene *cnd-1* in ‘D’ subtree and fitted curves. The X-axis represents time, the Y-axis represents gene expression. Points indicate the gene expression data, and lines represent the fitted curves, with colors indicating different cells

Furthermore, due to the varying lifetimes of individual cells, the time-series data are not pairwise, rendering the calculation of Pearson correlation coefficients infeasible. To address this challenge, a piecewise polynomial function *F*(*m*, *n*) called gene expression function is applied to fit tree-shaped gene expression data $\varvec{ts}(m, n)$. In detail, for five main cell lineage trees, denoted by ‘AB’, ‘C’, ‘D’, ‘E’, and ‘MS’, constrained linear regression is employed to fit gene expression data for each subtree. Specifically, for each cell, if the number of data points is less than 10, a 3-degree polynomial function is used to fit the data. For cells with at least 10 time points, the degree of the polynomial is increased by one for every *n* additional point, where *n* is chosen according to the Bayesian Information Criterion [23].During the process, constraints are imposed to ensure that the polynomial function is differentiable at each cell division point. The histogram of the coefficient of determination $R^2$ for a total of 870 fitted subtrees is shown in Fig. 3a, and an example of the fitting results for the ‘D’ lineage of gene *cnd-1* is shown in Fig. 3b.

Afterward, since fluorescent proteins are resistant to degradation in biological organisms, most gene expression data in cells does not decrease [6]. Hence cointegration effects might lead to excessively high Pearson correlation coefficients, making it challenging to identify the true clusters. Therefore, after the model is well-fitted,the gene expression rate function $F^{\prime }(m, n)$ can be obtained by taking the derivative of *F*(*m*, *n*) with respect to time. These functions can be converted to the same interval through translation and scaling, enabling the calculation of Pearson correlation coefficients. Additionally, a first-order difference is applied to $\varvec{ts}(m, n)$ to obtain gene expression rate data $\varvec{\Delta ts}(m, n)$. The steps of data preprocessing are detailed in Supplementary A.

### Biclustering model

In this paper, $\varvec{B_{k}} = \{ \varvec{G_{k}}, \varvec{C_{k}} \}$ represents the *k*-th biclustering submatrix of $\varvec{X}$, with dimension $m_{k} \times n_{k}$ and $k = 1, \cdots , K$. $\varvec{G_{k}} = \{ g_{k}^{1}, \cdots , g_{k}^{m_{k}} \}$ denotes the set of gene indices for the *k*-th biclustering, and $\varvec{C_{k}} = \{ c_{k}^{1}, \cdots , c_{k}^{n_{k}} \}$ represents the set of cell indices for the *k*-th biclustering. ${\textbf {Corr(}}\cdot , \cdot {\textbf {)}}$ denotes the Pearson correlation coefficient function, ${\textbf {KS(}}\cdot , \cdot {\textbf {)}}$ represents the *p*-value of the Kolmogorov-Smirnov (KS) test [24], ${\textbf {ES}}(\varvec{B_{k}})$ represents the gene expression size in the biclustering $\varvec{B_{k}}$. For the *k*-th ($k \ge 1$) biclustering, the objective function is defined as $f(\varvec{B_{k}})$, which can be expressed as follows:

1 $$\begin{aligned} \begin{aligned} f(\varvec{B_{k}}) =&\ \alpha \underbrace{\log \frac{1}{C_{m_{k}}^{2}n_{k}} \sum _{m<m^{\prime }} \sum _{n} {\textbf {Corr}}\left( F^{\prime }({g_{k}^{m}, c_{k}^{n}}), F^{\prime }({g_{k}^{m^{\prime }}, c_{k}^{n}})\right) }_{\text {gene correlation}} \\&+ \lambda \underbrace{\log \frac{1}{C_{n_{k}}^{2}} \sum _{n<n^{\prime }} \underset{m}{{\text {min}}} \{{\textbf {KS}}(\varvec{\Delta ts}({g_{k}^{m}, c_{k}^{n}}), \varvec{\Delta ts}({g_{k}^{m}, c_{k}^{n^{\prime }}}))\}}_{\text {cell similarity}}\\&+ \beta \underbrace{\log \left( {\textbf {ES}}(\varvec{B_{k}}) \right) }_{\text {gene expression size}}\\&- \delta \underbrace{\log \sum _{\begin{array}{c} g,c \in \varvec{B_{k}} \end{array}}\sum _{i=1}^{k-1} I _{\{ (g,c) \in \varvec{B_{i}} \}}}_{\text {overlap penalty term}} \end{aligned} \end{aligned}$$

where *m* and $m^{\prime } \in \left\{ 1, \cdots ,m_{k}\right\}$, *n* and $n^{\prime } \in \left\{ 1,\cdots ,n_{k}\right\}$. $\alpha$, $\lambda$, $\beta$, and $\delta$ are tuning parameters, and to prevent identifiability issues, $\beta$ was set to 1. In the last term, $\varvec{G_{0}} = \varvec{C_{0}} = \emptyset$. The goal is to use GA to sequentially detect biclusters that maximize ${{\text {argmax}}} \ f(\varvec{B_{k}})$.

It is essential to note that Formula (1) imposes the requirement that the biclustering must have at least 2 rows and 2 columns to be meaningful. Formula (1) comprises four indices representing four key components: gene correlation, cell similarity, gene expression size, and overlap penalty term. Below, a detailed explanation of the motivations and meanings of the four indices will be provided.

#### Gene correlation

The Pearson correlation coefficients between genes are computed across all cells. Due to the presence of multiple copies of the same gene across multiple measurements, relatively high correlation is typically observed among these different gene copies. Additionally, higher correlation is observed among some genes with similar or related functions [25], and it is expected that these genes will be grouped into the same cluster. Therefore, the consideration of the correlation between genes is incorporated into Formula (1). Here, the correlation between genes is measured by the Pearson correlation coefficient, which quantifies the linear association between genes. The definition of gene correlation is as follows. First, calculate the correlation coefficient between the expression rate functions of two genes within a single cell. Second, take the average of the correlation coefficients for these two genes across all cells within the bicluster. Finally, calculate the correlation coefficients among all gene pairs within the bicluster using the aforementioned steps and take the average. Details can be found in Supplementary B.

#### Cell similarity

After differentiating $\varvec{ts}(m, n)$ to obtain $\varvec{\Delta ts}(m, n)$, it is observed that the distribution of $\varvec{\Delta ts}(m, n)$ for cells within the same lineage is similar. Hence, the KS test is employed to assess differences in data distribution and examine the similarity in gene expression rata data. In KS test, the null hypothesis assumes that two samples are drawn from the same distribution. The *p*-value obtained from KS test serves as a metric to measure cell similarity, with higher *p*-values indicating that cells are more similar in terms of data distribution. The corresponding heatmap of KS test *p*-values between 145 known cell fates can be found in Figure S3 of Supplementary C, and the definition of cell similarity is as follows. First, conduct a KS test on the rate data of two cells within a single gene to obtain a *p*-value. Second, take the minimum *p*-value obtained for these two cells across all genes within the bicluster. Finally, use the aforementioned steps to calculate *p*-values for all pairs of cells within the bicluster and take the average.

#### Gene expression size

Based on the matrix $\varvec{Y}$ obtained in preprocessing, the gene expression 0-1 matrix $\varvec{Y_{k}}$ for genes and cells in the corresponding bicluster $\varvec{B_{k}}$ is obtained. The gene expression size is defined as the number of entries 1 in $$\varvec{Y_{k}} = \sum _{\begin{array}{c} g,c \in \varvec{B_{k}} \end{array}}y_{gc}$$. In the process of searching for biclusters, on one hand, it is essential to incorporate as many relevant key genes and cells as possible. On the other hand, there is a requirement to expedite convergence to some extent. Therefore, the gene expression size within biclusters is introduced as a key factor in the objective function.

#### Overlap penalty term

Considering that certain genes or cells may play different roles in distinct biological processes, allowing a certain degree of overlap between biclusters may be more biologically plausible. However, to prevent the discovery of highly repetitive biclusters, an overlap penalty term is introduced into Formula (1). This term penalizes the intersection of genes and cells between the current bicluster $\varvec{B_k}$ and the preceding $\varvec{B_{k-1}}$ biclusters, with the aim of restricting overlap between biclusters. When searching for the first bicluster, the value of the overlap penalty term is set to 0.

**Algorithm 1:**
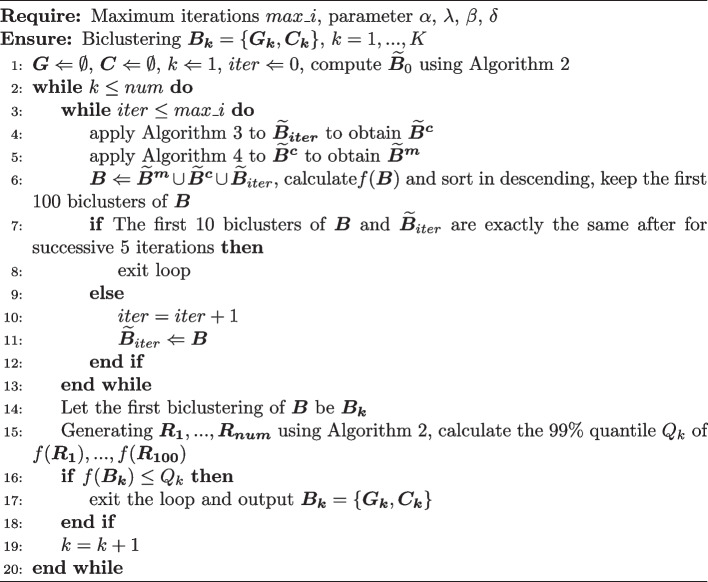
Biclustering search algorithm

**Algorithm 2:**
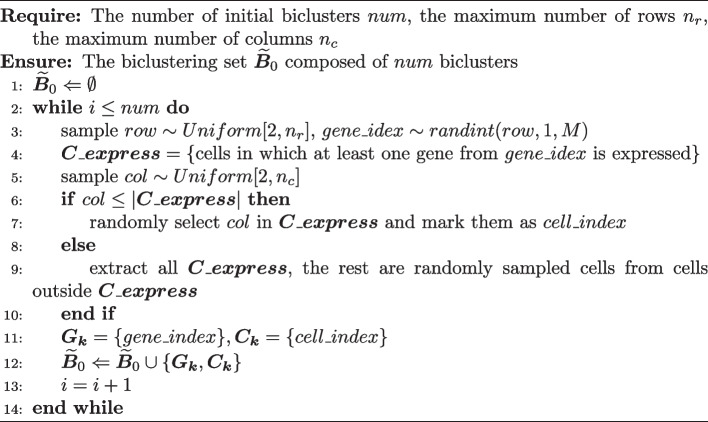
Initialization algorithm

**Algorithm 3:**
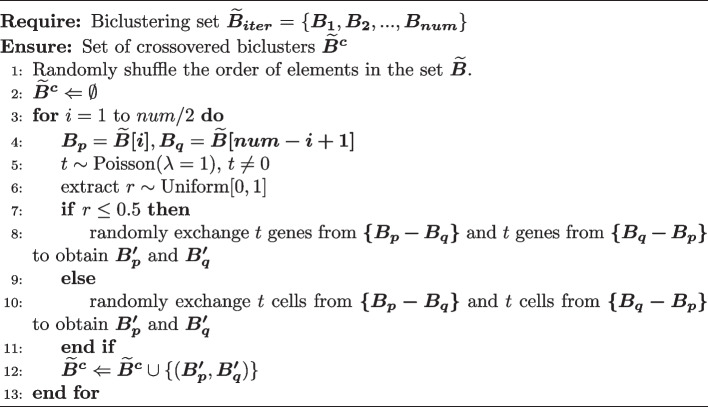
Crossover algorithm

**Algorithm 4:**
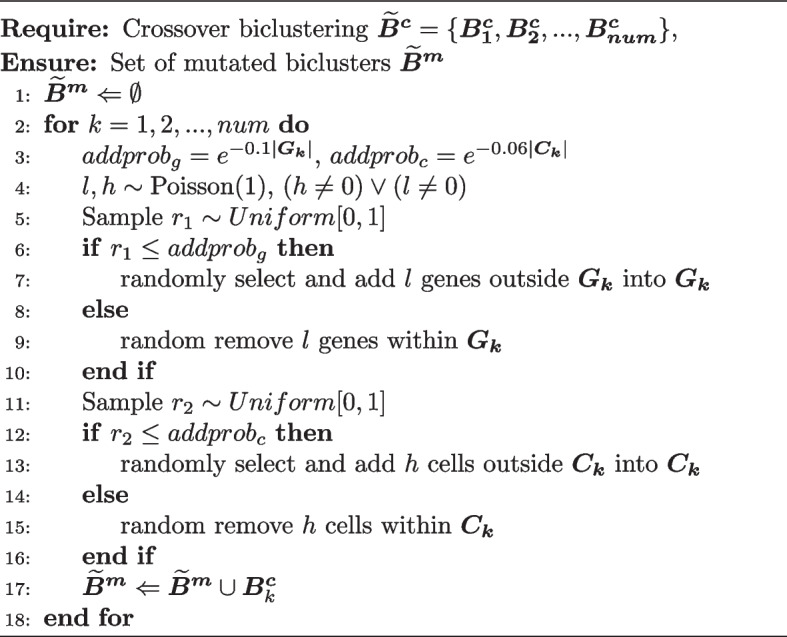
Mutation algorithm

### Genetic algorithm

GA is primarily employed to sequentially detect biclusters that maximize the objective function. The core of this GA is the biclustering search algorithm, and the specific process is outlined in Algorithm 1.

The biclusters are first initialized as detailed in Algorithm 2. The underlying idea is to random generate an initial set of biclusters with relatively large gene expression size. Our approach involves first sampling gene indices and then cell indices. This sequencing of sampling helps eliminate cells that lacked gene expression, thereby resulting in initial biclusters with larger gene expression sizes. This approach contributes to expediting the convergence speed of Algorithm 1 to some extent.

In each iteration, crossover is performed as described in Algorithm 3. The idea is to randomly pair biclusters and select a certain number of genes or cells to exchange within each pair. Then, mutations are applied to them, as outlined in Algorithm 4. The fundamental idea is to randomly add or remove genes or cells within the bicluster. At the end of Algorithm 3 and Algorithm 4, it is checked whether the gene expression proportions of each row and column are less than a certain threshold (set to 0.6). If the gene expression proportion is below the threshold in any row or column, the rows or columns with the smallest gene expression proportions are iteratively removed until no row or column within the bicluster has a gene expression proportion below the threshold. This operation helps improve the quality of the bicluster population, which accelerates the convergence of the objective function towards the maximum value.

The number of biclusters, denoted as *K*, is determined using the following method, serving as the stopping criterion mentioned in Algorithm 1: Calculate the 99% quantile $Q_{k}$ of the 100 submatrices randomly generated by Algorithm 2 in searching for *k*-th bicluster, and the objective function value $f(\varvec{B_{k}})$ for the candidate bicluster $\varvec{B_{k}}$. If $f(\varvec{B_{k}}) \le Q_{k}$, then stop searching. The hyperparameter settings for all algorithm are shown in Table 1.

**Table 1:** Hyperparameters in biclustering model search algorithm

Algorithm	Hyperparameter	Dataset
1	$max\_i$	1000
	$\alpha$	0.55
	$\lambda$	0.25
	$\beta$	1
	$\delta$	0.065
2	*num*	100
	$n_r$	10
	$n_c$	15

## Results

### Clustering analysis

First, cells are clustered using traditional clustering. Specifically, hierarchical clustering [26] is employed based on the preprocessed gene expression 0-1 matrix $\varvec{Y}$. The number of clusters is determined with the assistance of the Adjusted Rand Index [27]. The results of cell clustering are detailed in Supplementary D. The distribution of cell names displayed in the results indicates a certain level of consistency within the same cluster, while cell names in different clusters exhibit noticeable differences. Moreover, from a cellular fate perspective, most cells within the majority of clusters exhibit relatively pure cell fates.

Therefore, it is believed that the results of cell clustering are meaningful, as gene expression patterns vary among different cell clusters, and these variations might be overlooked by directly clustering genes. To validate this idea, a comparison of two gene clustering approaches is conducted. The first one involves gene clustering using all cells as features, while the second one involves separate gene clustering within each cell cluster obtained above. The clustering results are provided in Supplementary D, and large differences are observed between these two clustering results. Therefore, during the gene clustering process, it is necessary to consider the impact of heterogeneity among cells. This consideration is also the inspiration behind the adoption of biclustering algorithms in this study.

### Toy example analysis

Before this, ablation experiments are conducted on the objective function using toy examples, focusing on three components: gene correlation, cell similarity, and gene expression size. These experiments fail to produce satisfactory biclustering results and all ablation experiment results are included in the Supplementary F. Upon removing gene correlation, it is found that different copies of the same gene are barely clustered together within the same biclusters, and there is almost no evident regulatory relationship between different genes. Removing cell similarity results in a diverse range of cell fates within the same biclusters. Removing gene expression size results in a significant decrease in the number of genes and cells within biclusters, and most genes and cells lack apparent correlations.

The feasibility and effectiveness of TSBic are validated using a small-scale dataset. Genes with three or more copies are selected out, including 51 copies from 13 different genes, and 145 cells with known cell fates, covering a total of 10 cell fates. The results are evaluated according to the criterion that different copies of the same gene should be clustered to the same bicluster, and cells within the same lineage or share the same cell fate should be clustered to the same bicluster.

**Fig. 4:**
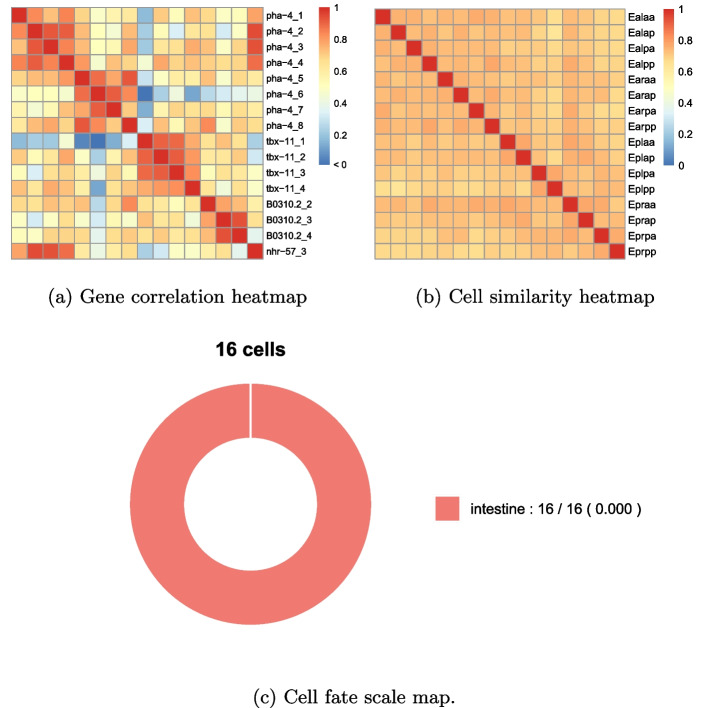
The first biclustering result of toy example analysis. **a** Heatmap of Pearson correlation coefficient matrix between genes. **b** Heatmap of KS test *p*-values matrix between cells. **c** Cell fate proportion diagram. Colors represent cell fates, sector area represents proportions, and the legend indicates cell fate names. The number before “/” represents the number of cells with the specified fate within that cluster, and the number after “/” represents the total number of cells with that fate. The value in parentheses indicates the *p*-value of cell fate enrichment analysis for that fate

Algorithm 1 is applied to the small-scale dataset, with a total runtime of 45 h. The memory usage during execution on the server is 1.2GB. As a result, a total of nine biclusters are obtained. Gene correlation heatmaps, cell similarity heatmaps, and cell fate scale maps are demonstrated for each bicluster. Only the first biclustering result is showcased in Fig. 4, while the remaining biclustering results can be found in Supplementary F.

From Fig. 4, the first identified bicluster comprises 16 copies and 16 cells, where all the copies of gene *pha-4* and *tbx-11* are included. For gene *B0310.2*, except for one copy that isn’t detected due to relatively low expression size, all other copies are present in the bicluster.

In this bicluster, based on gene information retrieved from WormBase [25], it is found that gene *pha-4* and *nhr-57* are both expressed in the intestine, and an indirect regulatory relationship exists between gene *pha-4* and *tbx-11*. Specifically, according to the biomedical interaction repository BioGRID [28], gene *pha-4* is regulated by *pop-1*, and gene *pop-1* is regulated by *tbx-11*. Furthermore, all 16 cells within the first bicluster belong to the ‘intestine’ fate. Actually, the majority of discovered biclusters reveal that different copies of the same gene, as well as cells with the same cell lineage or cell fate, belong to the same bicluster. In summary, the experimental results on the small-scale dataset validate the feasibility and effectiveness of the proposed biclustering model.

In addition, more experiments are completed. On one hand, three classical biclustering models are utilized, including the CC model [15], the plaid model [17], and the xMOTIFs model [29]. On the other hand, a comparison is conducted with two recently developed biclustering models: the QUBIC2 model [21] and the ARBic model [22]. In the context of these biclustering models, multiple experiments are conducted with different parameter settings and random seeds. However, the CC model and xMOTIFs model do not identify any biclusters, while three biclusters are detected by the plaid model, four biclusters are detected by the QUBIC2 model, and five biclusters are detected by the ARBic model. In these biclusters, the expression of genes is minimal or virtually absent in certain cells. Additionally, many cells from the same lineage are omitted, and there is a mixture of cells from different lineages, including cells with different cell fates. The specific details of these biclusters can be found in the Supplementary G.

Additionally, the computational time and memory usage of these algorithms are being considered. In terms of computational time, the TSBic method has an average running time of 45 h on this dataset, outperforming the CC (60 h), ARBic (65 h), and xMOTIFs models (72 h) but being lower than the Plaid model (42 h) and the QUBIC2 model (38 h) in terms of efficiency. In terms of memory usage, the TSBic method occupies 1.2GB of memory, less than that of QUBIC2 (1.4GB), ARBic (1.4GB), and xMOTIFs models (1.5GB), but more than the Plaid model (0.5GB) and the CC model (0.7GB).

### Complete real data analysis

The complete dataset consists of 174 copies (including 104 different genes) and 724 cells (including 145 cells with known cell fates). For the complete dataset, some minor modifications are made to the original biclustering algorithm. In detail, all copies corresponding to the same gene are added to or removed from the bicluster as a whole. To this end, for gene correlation, the correlation coefficient of two genes within the bicluster is defined as the average of correlation coefficients between pairwise copies. For cell similarity, the similarity between two cells is defined as the minimum *p*-value from the KS test conducted on all gene copies between these two cells.

**Fig. 5:**
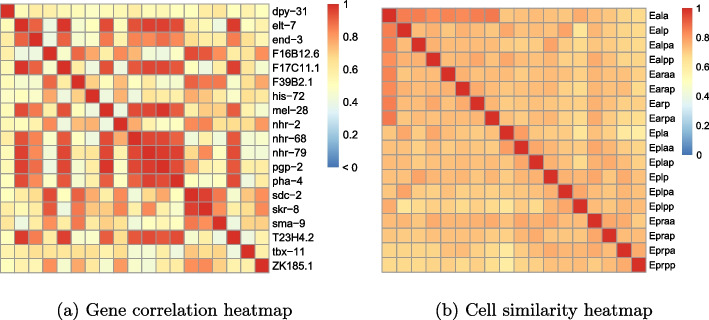
The first biclustering result of the complete real dataset. **a** Heatmap of Pearson correlation coefficient matrix between genes. **b** Heatmap of KS test *p*-value matrix between cells

The Algorithm 1 is applied to the complete dataset, with a total runtime of 145 h. The memory usage during execution on the server is 1.5GB. A total of ten biclusters are detected and only the first bicluster with 19 genes and 18 cells are presented, including the gene expression heatmap and cell similarity heatmap as shown in Fig. 5. Due to the fact that only about 20% of cells in the complete real data have known cell fate information, it is not feasible to observe the proportion of cell fate in the biclusters. Therefore, the results on the complete data do not include the cell fate scale map. The remaining biclustering results can be found in Supplementary H.

Based on the obtained biclustering results, an evaluation is conducted from both the gene and cell perspectives. On one hand, the cell clustering results are assessed based on the cell lineages. It can be observed that, similar to the first bicluster in the toy example analysis, the cells in the first bicluster are all from the ‘E’ lineage. The other biclustering results also show cases where multiple cell lineages cluster together within a cluster. For instance, in the second bicluster, most cells are from the ‘AB’ lineage, but there are also cells from the ‘C’ and ‘MS’ lineages. The majority of cells from the ‘C’ and ‘MS’ lineages are found to share a fate associated with dermal tissue, aligning with the fate of cells from the ‘AB’ lineage. In fact, ‘E’ lineage cells are mainly concentrated in the first and seventh biclusters; ‘C’ lineage cells are mainly concentrated in the eighth and tenth biclusters; ‘MS’ lineage cells are mainly concentrated in the fifth and ninth biclusters; ‘AB’ lineage cells are distributed in the remaining biclusters. These findings indicate that most of the biclusters we found have cell clustering results that are consistent with the biological backgrounds.

**Fig. 6:**
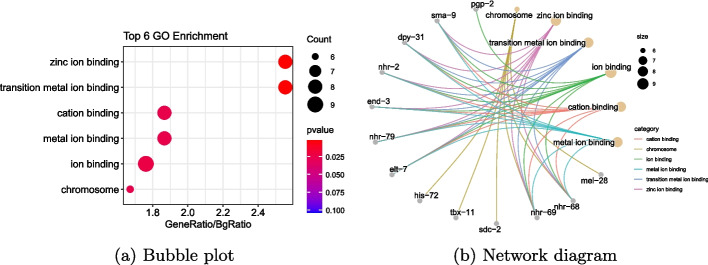
Gene enrichment analysis results. **a** Gene enrichment analysis bubble plot. The X axis represents the enrichment multiple, and the Y axis represents the name of the enriched pathway. The size of the bubble indicates the number of genes enriched, and the color of the bubble indicates the *p*-value of gene enrichment analysis. **b** Gene network diagrams for each pathway. The gray dot represents the name of the enriched gene in the class, the yellow dot indicates the name of the enriched pathway, with the size indicating the number of enriched genes. The lines connect each pathway with its enriched genes, and the color indicates the category of the pathway

On the other hand, gene enrichment analysis is employed to evaluate the clustering results of genes in biclusters, according to the Gene Ontology annotation database [30]. To present the results, bubble diagrams showcasing the most prominent pathways and gene network diagrams for each pathway are applied. In the main text, the results of gene enrichment analysis for the first bicluster are displayed in Fig. 6. The remaining biclustering results can be found in the Supplementary H.

From Fig. 6a, it is observed that several pathways are significantly enriched at a significance level of 0.05. In fact, within the first bicluster, eight genes *elt-7*, *end-3*, *dpy-31*, *nhr-2*, *nhr-68*, *nhr-69*, *nhr-79*, and *sma-9* demonstrate enrichment in the zinc ion binding process at a highly significant *p*-value of 0.002. This highlights that the gene biclustering outcomes hold substantial biological significance within the context of molecular function and cell component [31]. Furthermore, based on the existing research results on gene regulatory relationships from BioGRID [28], it is found that in the first bicluster, genes *elt-7*, *end-3*, *nhr-2*, *nhr-79*, *pha-4*, and *tbx-11* exhibit DNA-binding transcription factor activity and RNA polymerase II-specific activity. They play important roles in gene transcription regulation and influence cellular function by regulating gene transcription mediated by RNA polymerase II. Genes *nhr-2*, *nhr-79*, and *tbx-11* are involved in the regulation of cell fate and transcriptional control, functioning in cell differentiation and specialization processes, thereby affecting cell function and fate by regulating the transcription of specific genes. Genes *elt-7*, *end-3*, *nhr-2*, *nhr-79*, *pha-4*, *tbx-11*, and *T23H4.2* are expressed in the cell nucleus and are involved in regulating RNA synthesis and gene transcription. They play crucial roles in regulating gene expression within cells, thereby influencing cell function and characteristics. The findings affirm that a majority of the biclustering results discovered in this study carry significant biological relevance, effectively capturing the spatiotemporal expression patterns among genes within different cells.

## Discussion and conclusion

In this paper, a biclustering model TSBic is proposed based on the tree-shaped single-cell gene expression data in *C.elegans*, and the biclusters are detected by Genetic Algorithm through maximizing the specially designed objective function. Gene enrichment analysis evaluates the obtained biclusters, and the results indicate that most of the gene and cell biclusters discovered exhibit meaningful biological relevance and importance. These findings affirm the effectiveness of the proposed method.

Although our study has yielded some meaningful results, there are still that could be further improved. First, this study introduces a constrained piecewise polynomial function to address the issue of non-pairwise data when fitting gene expression data. In this process, the fitting may not be well enough, especially for cells with shorter lifetimes. Therefore, further exploration is needed to investigate fitting function forms that better match the data, aiming to enhance the accuracy of the fitting. Second, Genetic Algorithms are computationally intensive methods, and the size and dimensions of the data may pose challenges in terms of computational complexity. Further exploration of optimization methods is needed to accelerate the convergence of algorithm and handle large-scale dataset.

### Electronic supplementary material

Below is the link to the electronic supplementary material.

Supplementary file 1 (PDF 2722kb)

## Data Availability

The datasets used in this study can be obtained from http://epic.gs.washington.edu/.

## Code availability

The source codes of TSBic are available at https://github.com/Guanqi0827/TSBic
