# Supplementary material for: Biclustering analysis on tree-shaped time-series single cell gene expression data of Caenorhabditis elegans
Source: BMC Bioinformatics. 2024 May 9;25:183. doi: 10.1186/s12859-024-05800-y (PMC11080145; doi:10.1186/s12859-024-05800-y)
Supplement: Supplementary file 1 — (PDF 2722kb) [file 12859_2024_5800_MOESM1_ESM.pdf]

Supplemental material for biclustering analysis on tree-shaped time-series single cell gene expression data of *Caenorhabditis elegans*

Qi Guan, Xianzhong Yan, Yida Wu, Da Zhou, Jie Hu
School of Mathematical Sciences, Xiamen University, Xiamen, Fujian, 361005, China

Contents

A Data preprocessing 2

B Calculation of Pearson correlation coefficient of fitted data 4

C Measuring cell similarity with KS test p-values 6

D Clustering analysis 7

E Ablation study 13

F Biclustering results on toy example 29

G Comparison with other biclustering models 37

H Biclustering results on the complete real data 50

A Data preprocessing

The real dataset used Murray et al. (2012) can be downloaded from `http://epic.gs.washington.edu/`. For each 4D confocal laser scanning microscopy experiment, there is a data file that contains the time series of each embryonic cell from birth to division or death. Each measurement represents the fluorescence protein intensity at a specific time point during the cell's lifetime. Due to the limited expression of certain genes or the late birth of some cells during the experimental process, there are missing values in the experimental data. Therefore, missing data is first detected, as specifically defined in Section 2.1 of the manuscript. For each gene corresponding to each cell in the real dataset, calculate the proportion of missing values. The histograms illustrating the proportions of missing values for genes and cells are shown in Figure S1. Recognizing that genes or cells with excessive missing values contribute little to biclustering, genes and cells with missing value proportions exceeding 60% are simultaneously removed.

After removing genes and cells with a significant amount of missing values, the matrix dimensions are reduced from 184×1083 to 174×724. In other words, biclustering is conducted on 174 copies (comprising 104 distinct genes) and 724 cells (including 145 cells with known cell fates). Furthermore, the results of gene expression onset times measured and provided by Hu et al. (2015) are utilized. Genes with expression in cells are marked as 1, while genes without expression are marked as 0, thereby constructing the gene expression 0-1 matrix $\boldsymbol{Y}$.

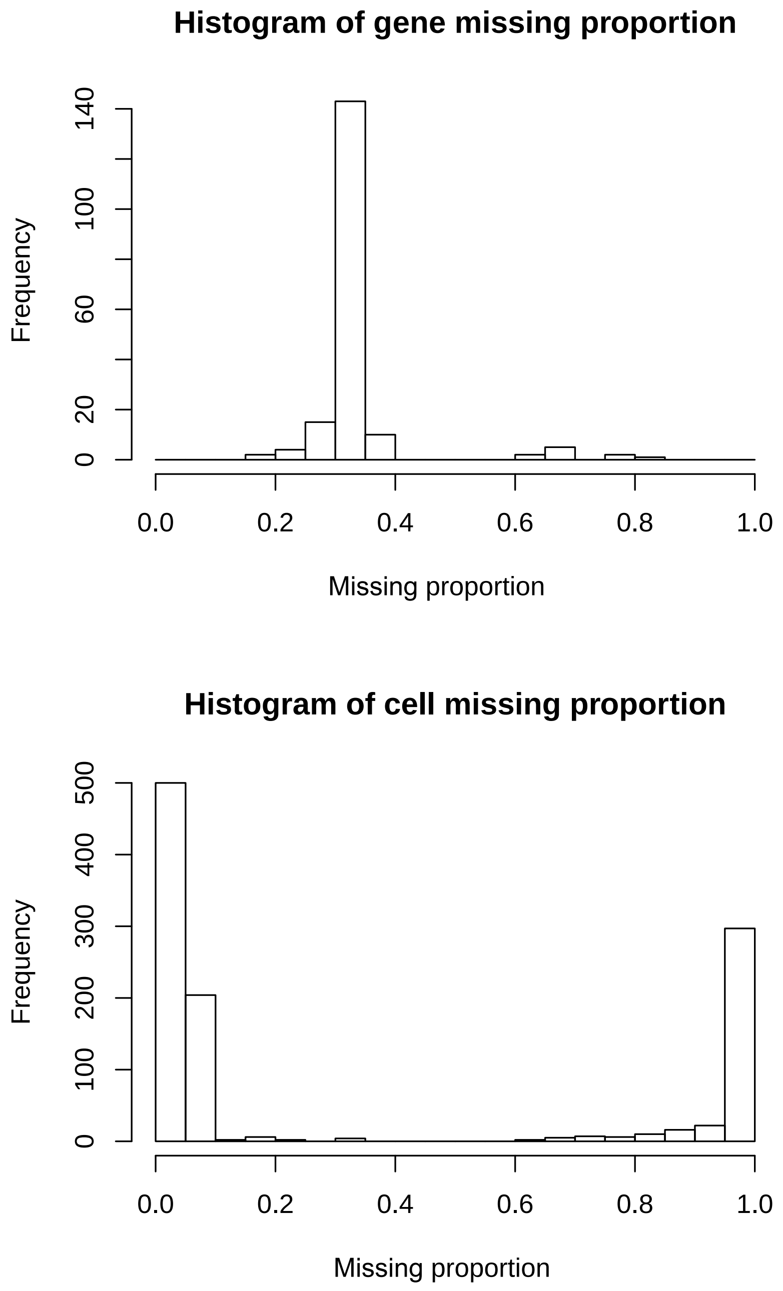

Figure S1: Histograms of missing value proportions for genes and cells in the raw data matrix. The X-axis represents the proportion of missing values, and the Y-axis represents the frequency.

B Calculation of Pearson correlation coefficient of fitted data

The Pearson correlation coefficients between the expression rate functions $F'(m, n)$ of individual genes have been computed, and the results are presented in Figure S2. The gene correlation results for all genes across all cells are displayed in the figure. It is noteworthy that copies of the same gene exhibit relatively high correlation. Consequently, the Pearson correlation coefficient between genes is considered as a metric to be incorporated into the objective function of the biclustering model.

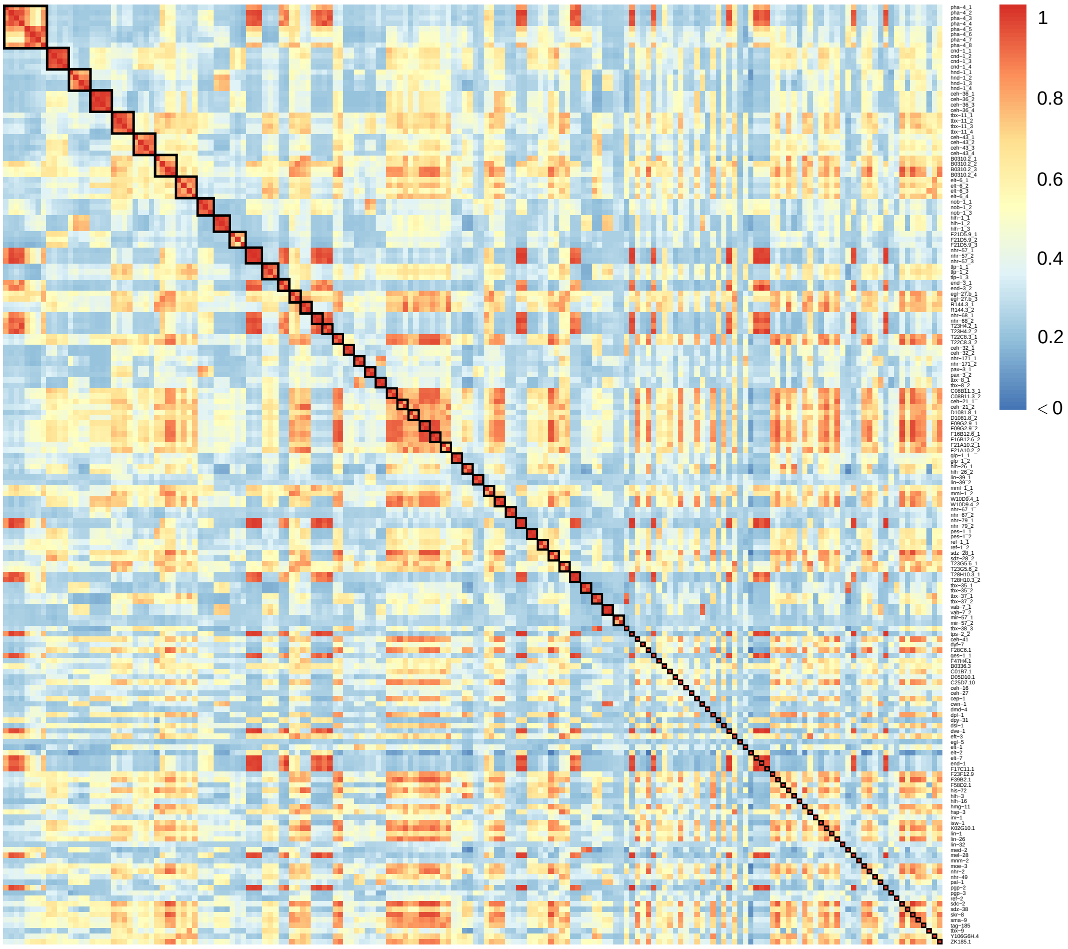

Figure S2: Heatmap of correlation coefficients matrix among gene expression profiles. Rows represent gene file names, with the gene name before the underscore "_" and the copy number of the file after the underscore "_". The black square contains different copies of the same gene.

Below, we will introduce the derivation process of the Pearson correlation coefficient between two functions. Let's begin by presenting the definition of the Pearson correlation coefficient. Consider a two-dimensional random variable (X, Y), with the conditions that

$Var(X) > 0$ and $Var(Y) > 0$. In this context:

$$Corr(X,Y) = \frac{Cov(X,Y)}{\sqrt{Var(X)}\sqrt{Var(Y)}}$$

is the Pearson correlation coefficient between X and Y.

In this study, it is assumed that both $f(T)$ and $g(T)$ are continuous functions, and T is assumed to follow a uniform distribution on the interval $[0,1]$. Employing the method for calculating expectations of random variable functions, the following can be derived:

$$E[f(T)] = \int_0^1 f(t)dt, \; E[f(T)^2] = \int_0^1 f(t)^2 dt,$$

$$E[g(T)] = \int_0^1 g(t)dt, \; E[g(T)^2] = \int_0^1 g(t)^2 dt,$$

$$E[f(T)g(T)] = \int_0^1 f(t)g(t)dt,$$

Hence

$$Var[f(T)] = E[f(T)^2] - (E[f(T)])^2,$$

$$Var[g(T)] = E[g(T)^2] - (E[g(T)])^2,$$

$$Cov(f(T), g(T)) = E[f(T)g(T)] - E[f(T)] \cdot E[g(T)],$$

The correlation coefficient between $f(T)$ and $g(T)$ can be calculated as follows:

$$Corr(f(T), g(T)) = \frac{Cov(f(T), g(T))}{\sqrt{Var[f(T)]}\sqrt{Var[g(T)]}}.$$

The gene expression rate functions $F'(m,n)$ mentioned in this paper can be easily transformed to domains $[0,1]$. Additionally, their definite integrals have analytical solutions. Therefore, the method introduced in this section can be used to compute the Pearson correlation coefficient based on gene expression rate functions.

C Measuring cell similarity with KS test p-values

After taking the difference of the time series of $\boldsymbol{ts}(m,n)$, it is observed that the $\boldsymbol{\Delta ts}(m,n)$ for most cells within the same cell fate are found to follow similar distributions. The Kolmogorov-Smirnov test p-values between individual cell $\boldsymbol{\Delta ts}(m,n)$ are calculated, and Figure S3 displays a heatmap of p-value similarities between 145 cells with known cell fates, where higher p-values indicate more similar distributions. Hence, the p-value from the KS test can be used as a metric for measuring the similarity between cells.

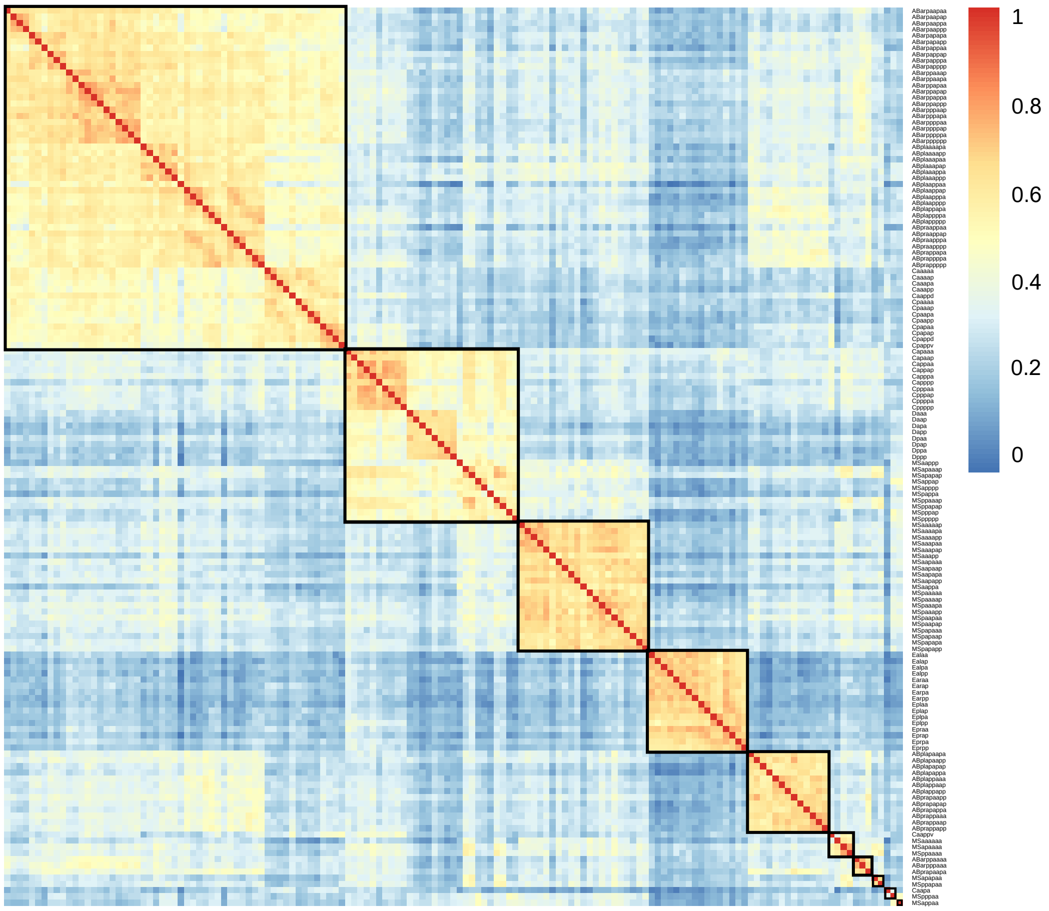

Figure S3: KS test p-value heatmap between cells. The black square represents cells with the same cell fate.

D Clustering analysis

In the gene expression 0-1 matrix $\boldsymbol{Y}$, 104 genes are represented by rows (different copies of the same gene are combined as a whole); columns represent 145 cells, with know cell fates. Before describing the specific process of cell clustering, let's first introduce the measures used to assess the quality of the clustering results.

(1) Cell Fate Enrichment Analysis: Analogous to gene enrichment analysis, we substitute cell fate for gene function. This essentially involves conducting a hypergeometric distribution hypothesis test.

(2) Cell Naming: As mentioned in Sulston et al. (1983), cell names are assigned based on lineage relationships. Therefore, cells with the same lineages are likely to belong to the same category.

Hierarchical clustering is employed, where the distance metric utilizes binary variable distance, and the similarity judgment utilizes the Ward method (Ward Jr, 1963). The number of clusters is determined with the assistance of the Adjusted Rand Index (Hubert and Arabie, 1985).

The dendrogram for cell clustering results is shown in Figure S4. From the distribution of cell names displayed in the figure, it is evident that cells within the same cluster exhibited a certain level of consistency in their names, while cells from different clusters showed distinct differences. As ABa cells differentiate into two distinct tissues, hypodermis and nerve tissue, and ABp cells differentiate into hypodermis and blast cells during the differentiation process, there are two clusters each for ABa and ABp. Furthermore, from the perspective of cell fate, most clusters consist of relatively pure cell fates. However, there are also some clusters where cell fates are not as pure. Taking cell cluster result ABp(2) branch in Figure S4 as an example, which is depicted in Figure S5, although this cluster encompasses multiple cell fates, these fates can be broadly categorized into two groups: neural tissue and pharyngeal tissue. Furthermore, the majority of these cell fates are supported by cell enrichment analysis.

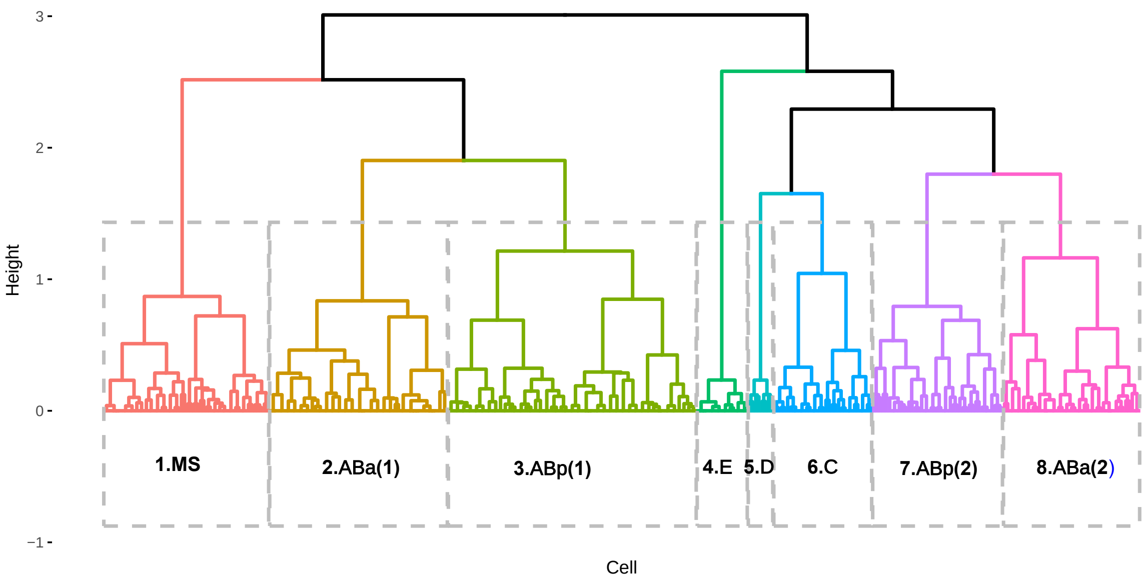

Figure S4: Cell clustering results dendrogram. Letters represent cell branch names, and colors represent different clusters.

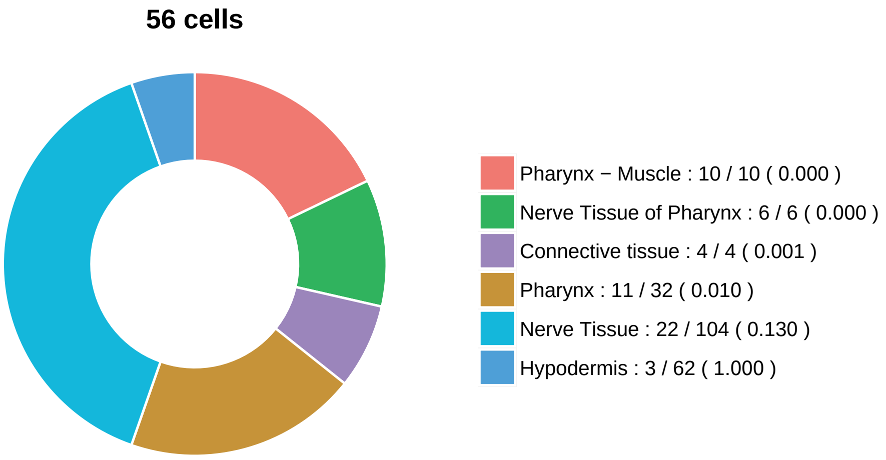

Figure S5: Pie chart illustrating the cell fate of ABp(2) branch in the cell clustering results. In the chart, colors represent different cell fates, and the size of each sector indicates the proportion. The legend denotes cell fates, where the number before "/" signifies the count of cells with that fate within the category, the number after "/" represents the total count of cells with that fate, and values in parentheses indicate the p-value of cell fate enrichment analysis.

In summary, the results indicate that gene expression may vary among different types of cells. Directly clustering genes using a one-way approach may overlook these differ-

ences, leading to suboptimal clustering results. To confirm this idea, a comparison is conducted of two gene clustering approach based on gene expression 0-1 matrix. The first one involves all cells, and the clustering results are shown in Figure S6. The second one is based on the cell clustering results obtained from Figure S4, where genes are independently clustered based on each cluster of cells. The clustering results for the eight clusters of cells are displayed in Figures S7-S14. Both methods employ hierarchical clustering for gene clustering, binary variable distance metrics, and the Ward method to measure similarity. Clearly, these two gene clustering approaches exhibit obvious differences. Therefore, during the gene clustering process, it is crucial to consider the impact of cell heterogeneity among different classes, which inspires the use of biclustering algorithms in this study.

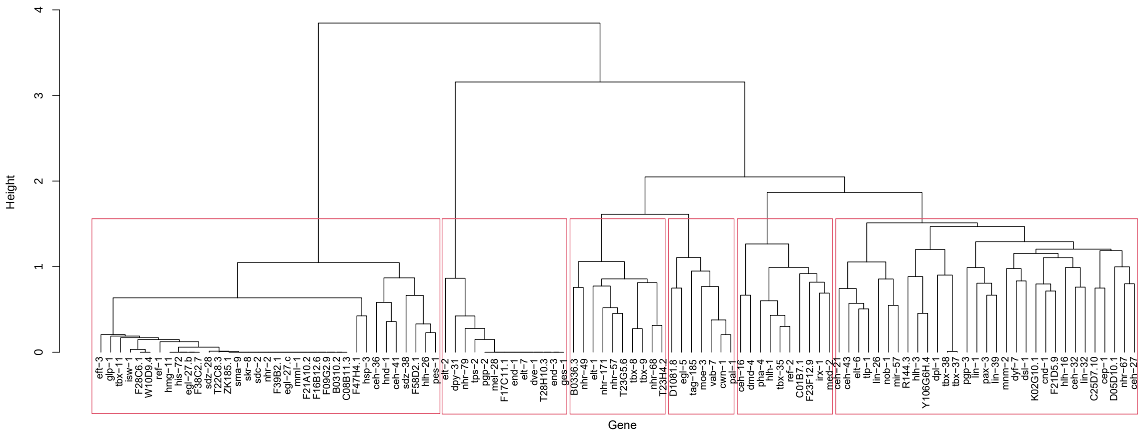

Figure S6: Hierarchical dendrogram of gene clustering for all cells. The horizontal axis represents gene names, and the red square represent different clusters.

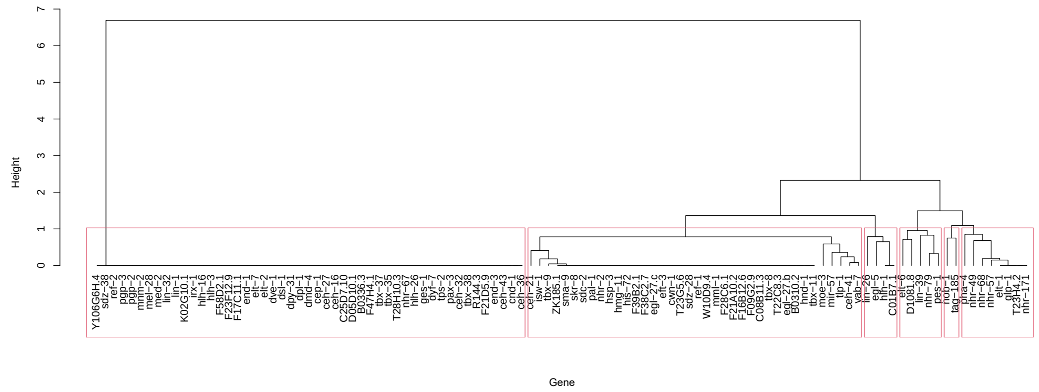

Figure S7: The dendrogram of gene clustering results for the ‘MS’ cluster of cells.

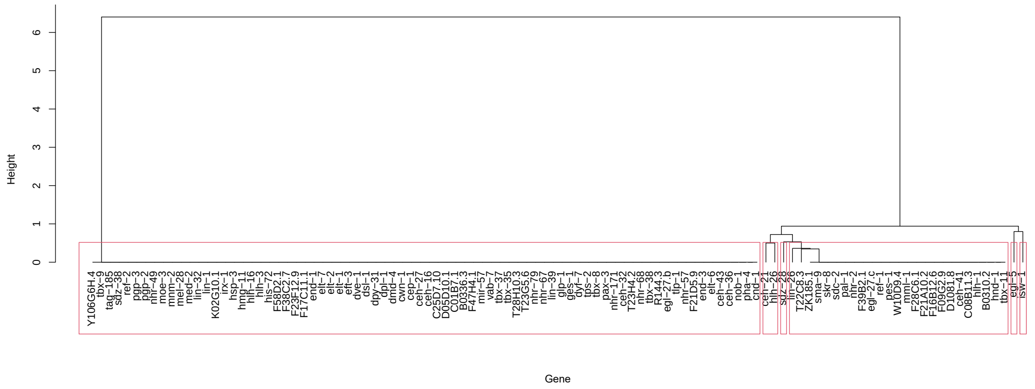

Figure S8: The dendrogram of gene clustering results for the 'ABa(1)' cluster of cells.

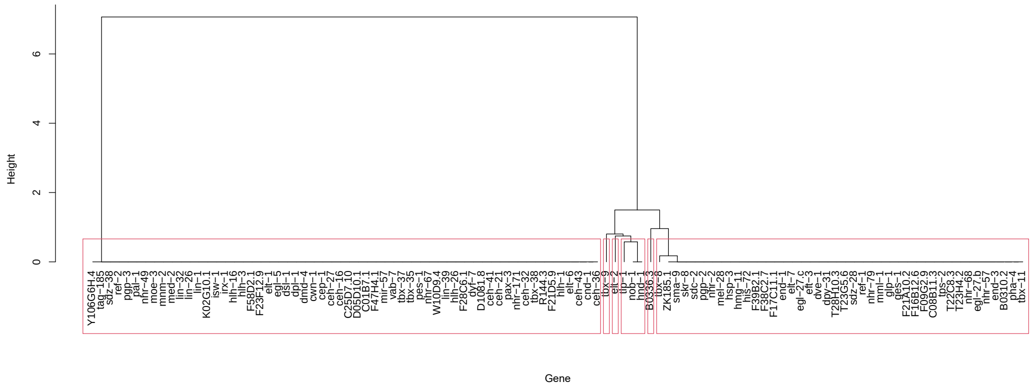

Figure S9: The dendrogram of gene clustering results for the 'ABp(1)' cluster of cells.

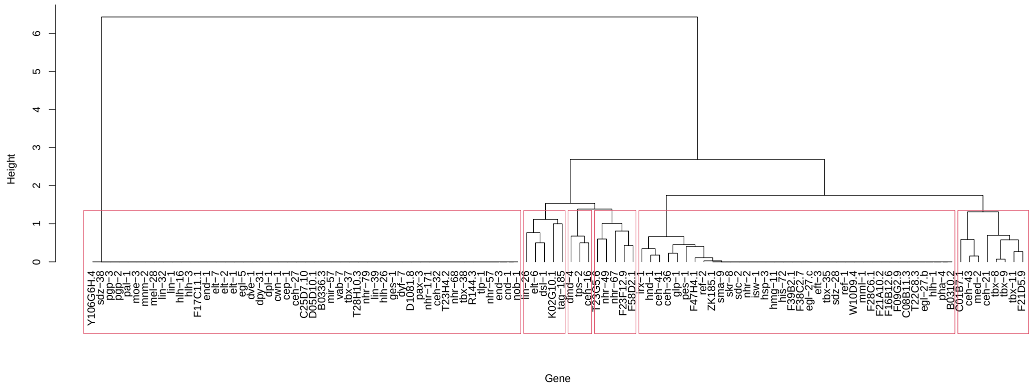

Figure S10: The dendrogram of gene clustering results for the 'E' cluster of cells.

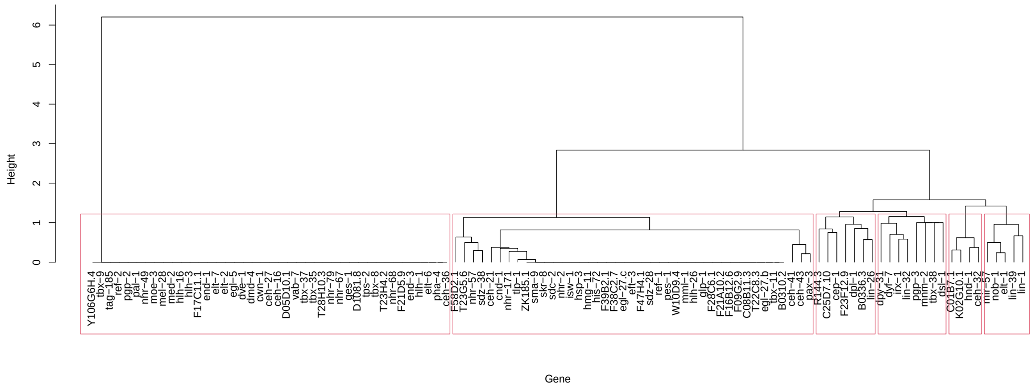

Figure S11: The dendrogram of gene clustering results for the 'D' cluster of cells.

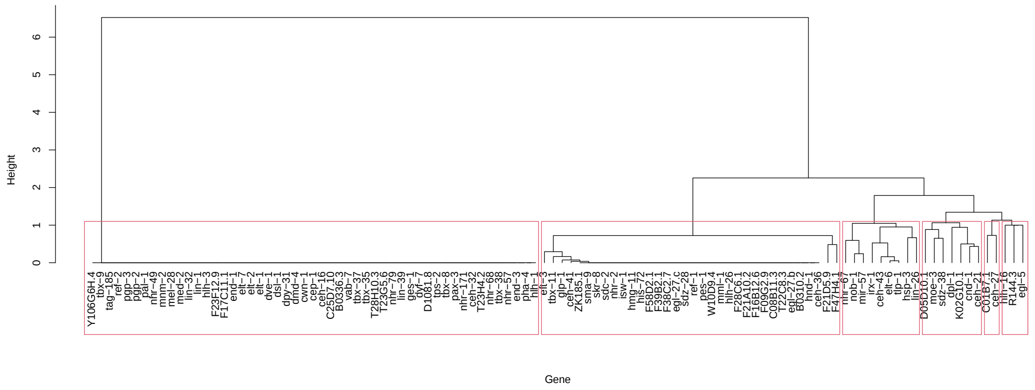

Figure S12: The dendrogram of gene clustering results for the 'C' cluster of cells.

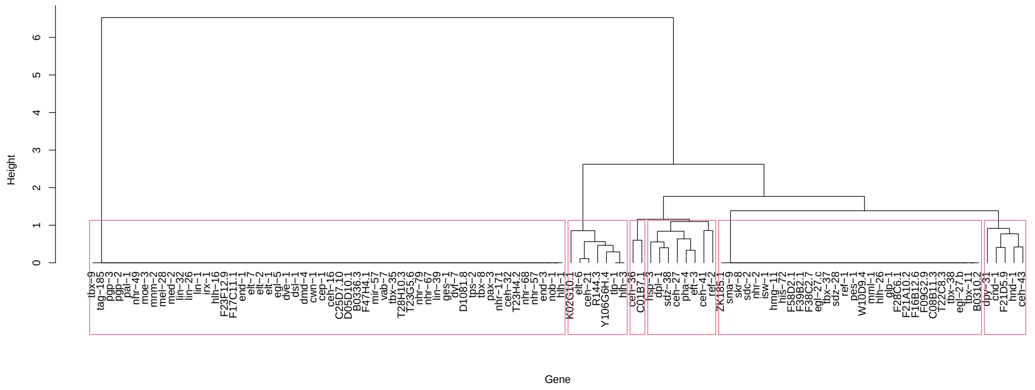

Figure S13: The dendrogram of gene clustering results for the 'ABp(2)' cluster of cells.

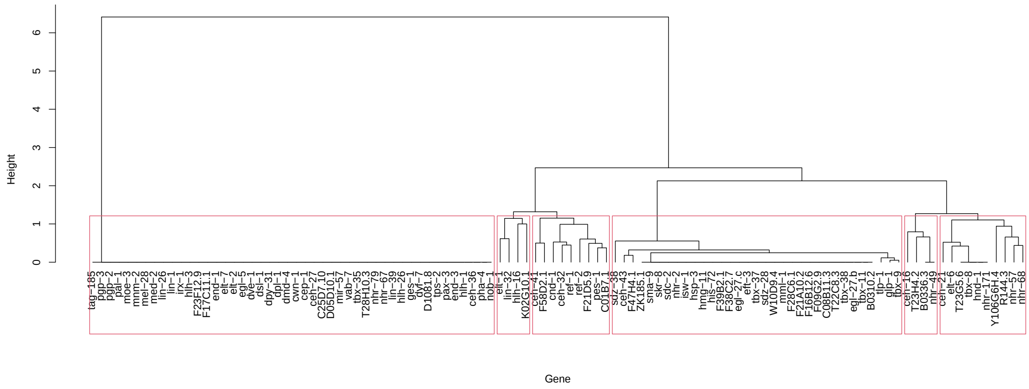

Figure S14: The dendrogram of gene clustering results for the ‘ABa(2)’ cluster of cells.

E Ablation study

To substantiate the importance of the components in our objective function, ablation experiments are conducted on the first three components (gene correlation, cell similarity, gene expression size) of the objective function on a toy example. Since removing the overlap penalty term will lead to duplicated outcomes, these three components are sequentially removed from the objective function, followed by the application of GA to obtain biclusters. Specifically, five, seven, and four biclusters are yielded, respectively, by the three components in the toy example. The specific results of the ablation experiments are as follows:

- The result after removing the gene correlation component:

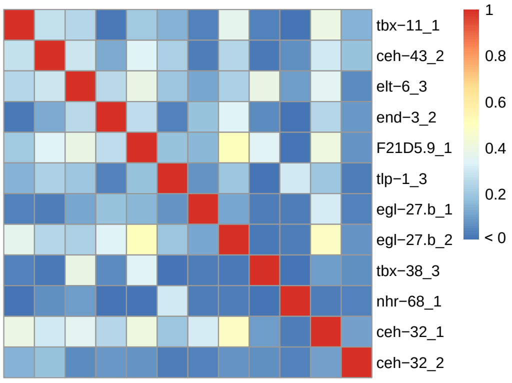

(a) Gene correlation heatmap

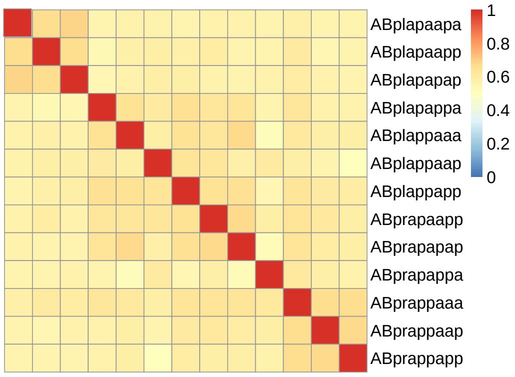

(b) Cell similarity heatmap

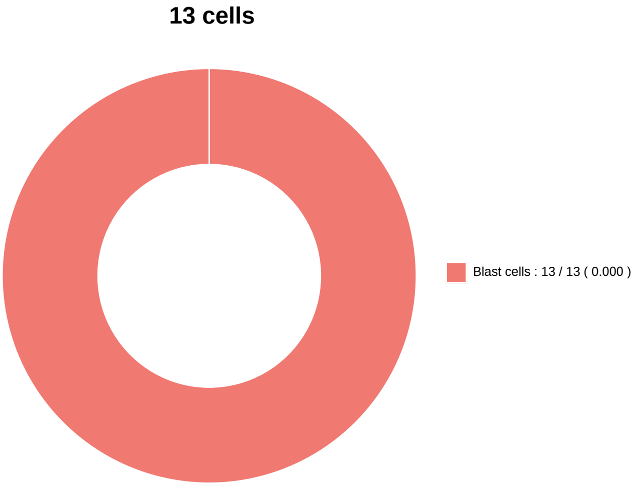

(c) Cell fate proportion plot

Figure S15: The first bicluster result obtained after removing the gene correlation component in the toy example. (a) Heatmap of Pearson correlation coefficient matrix between gene profiles. (b) Heatmap of KS test p-values matrix between cells. (c) Cell fate proportion diagram.

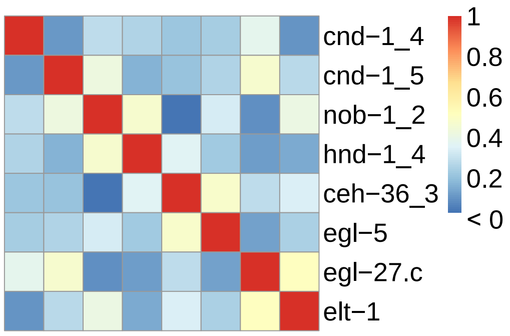

(a) Gene correlation heatmap

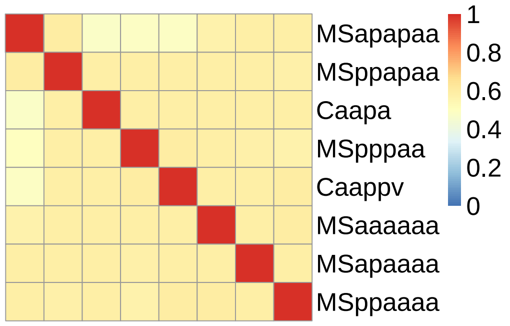

(b) Cell similarity heatmap

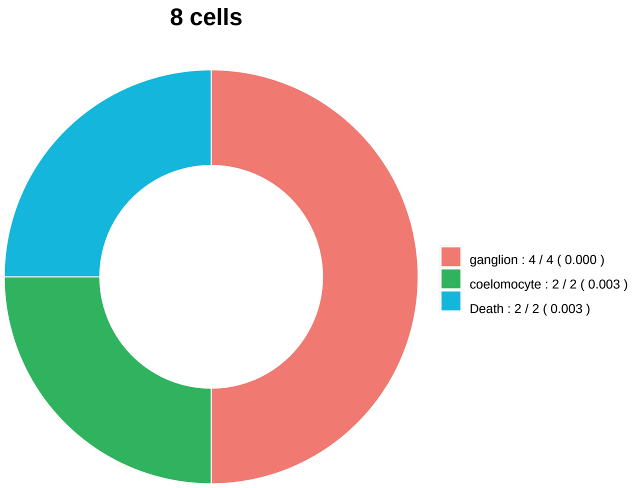

(c) Cell fate proportion plot

Figure S16: The second bicluster result obtained after removing the gene correlation component in the toy example. (a) Heatmap of Pearson correlation coefficient matrix between gene profiles. (b) Heatmap of KS test p-values matrix between cells. (c) Cell fate proportion diagram.

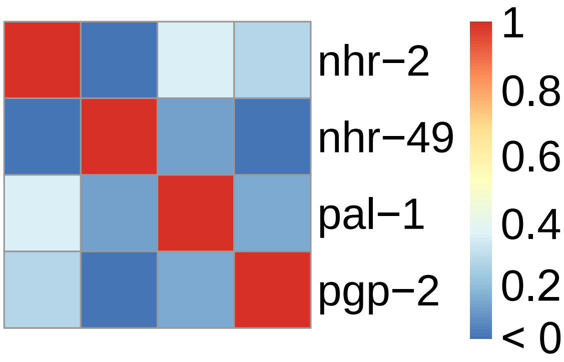

(a) Gene correlation heatmap

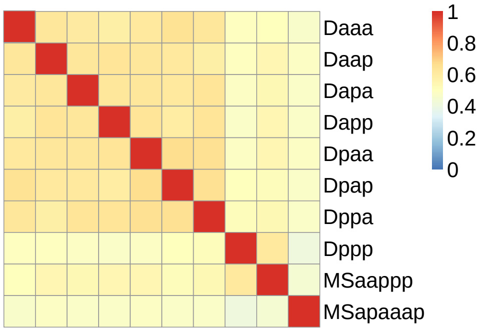

(b) Cell similarity heatmap

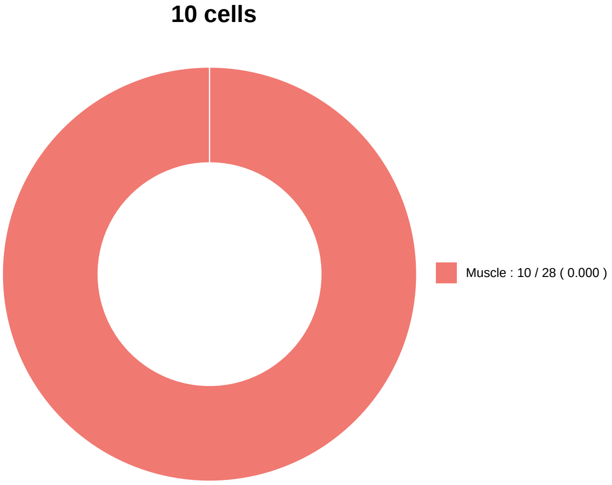

(c) Cell fate proportion plot

Figure S17: The third bicluster result obtained after removing the gene correlation component in the toy example. (a) Heatmap of Pearson correlation coefficient matrix between gene profiles. (b) Heatmap of KS test p-values matrix between cells. (c) Cell fate proportion diagram.

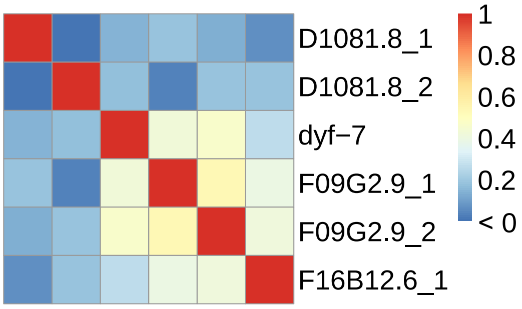

(a) Gene correlation heatmap

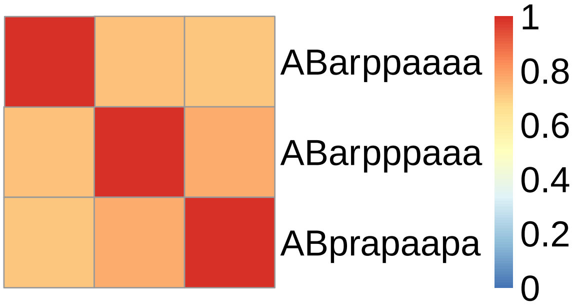

(b) Cell similarity heatmap

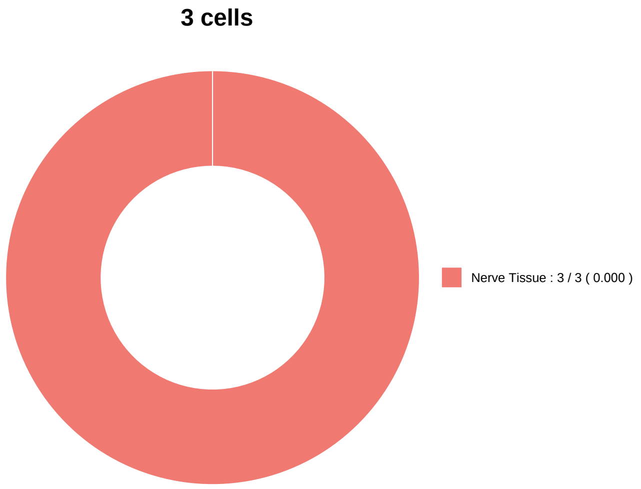

(c) Cell fate proportion plot

Figure S18: The fourth bicluster result obtained after removing the gene correlation component in the toy example. (a) Heatmap of Pearson correlation coefficient matrix between gene profiles. (b) Heatmap of KS test p-values matrix between cells. (c) Cell fate proportion diagram.

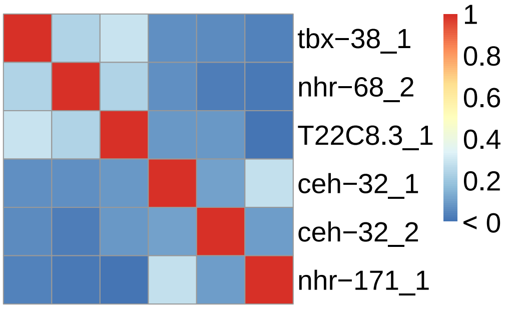

(a) Gene correlation heatmap

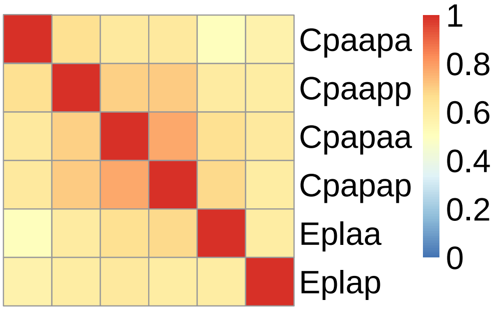

(b) Cell similarity heatmap

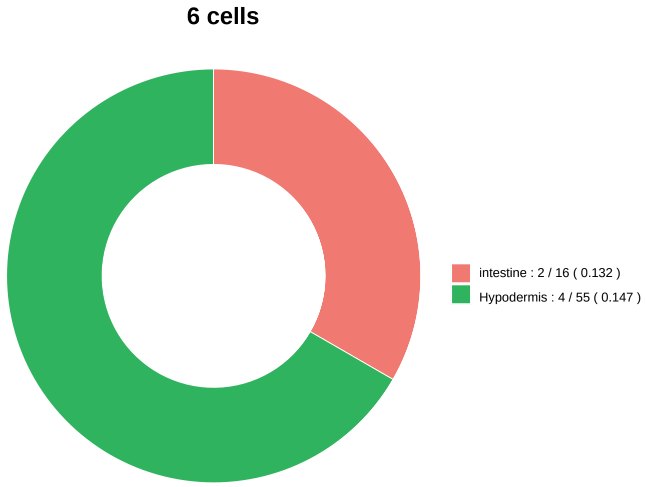

(c) Cell fate proportion plot

Figure S19: The fifth bicluster result obtained after removing the gene correlation component in the toy example. (a) Heatmap of Pearson correlation coefficient matrix between gene profiles. (b) Heatmap of KS test p-values matrix between cells. (c) Cell fate proportion diagram.

- The result after removing the cell similarity component:

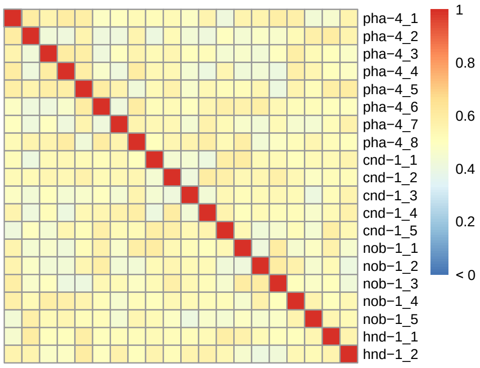

(a) Gene correlation heatmap

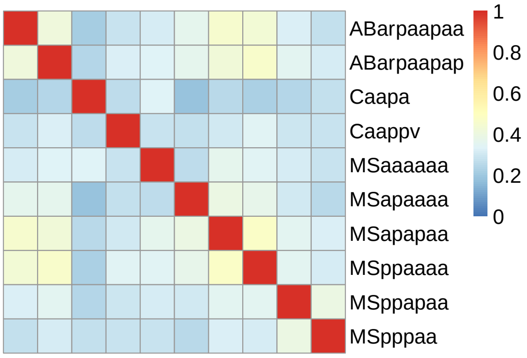

(b) Cell similarity heatmap

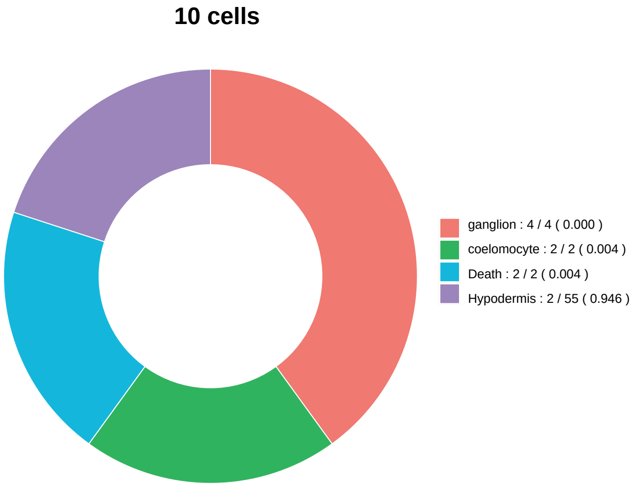

(c) Cell fate proportion plot

Figure S20: The first bicluster result obtained after removing the cell similarity component in the toy example. (a) Heatmap of Pearson correlation coefficient matrix between gene profiles. (b) Heatmap of KS test p-values matrix between cells. (c) Cell fate proportion diagram.

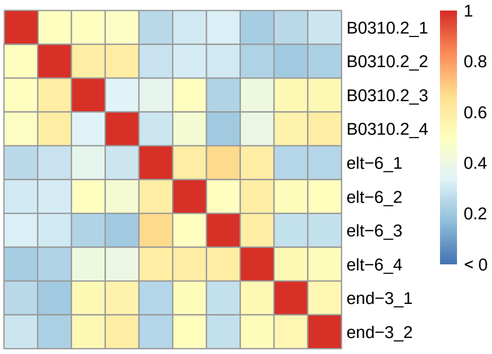

(a) Gene correlation heatmap

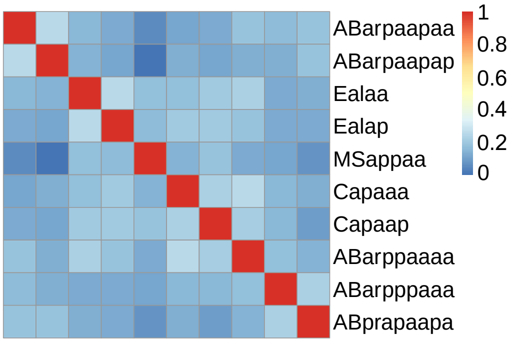

(b) Cell similarity heatmap

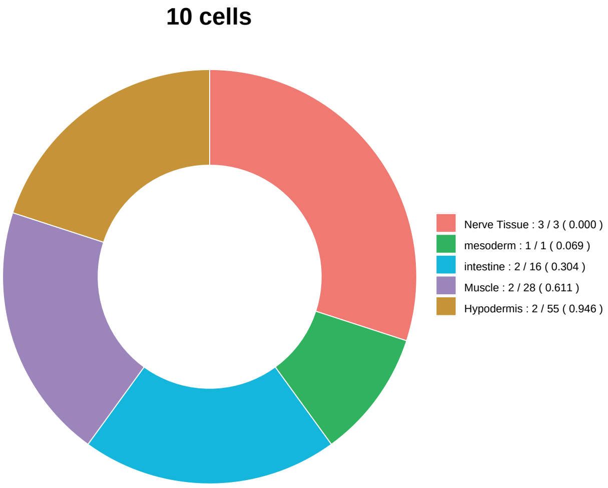

(c) Cell fate proportion plot

Figure S21: The second bicluster result obtained after removing the cell similarity component in the toy example. (a) Heatmap of Pearson correlation coefficient matrix between gene profiles. (b) Heatmap of KS test p-values matrix between cells. (c) Cell fate proportion diagram.

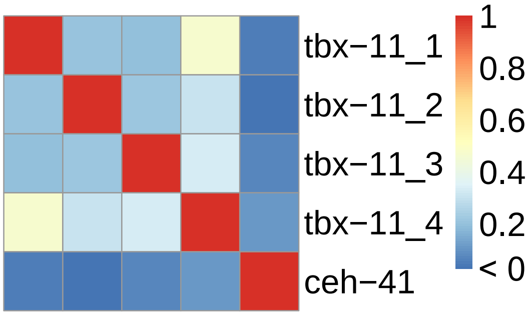

(a) Gene correlation heatmap

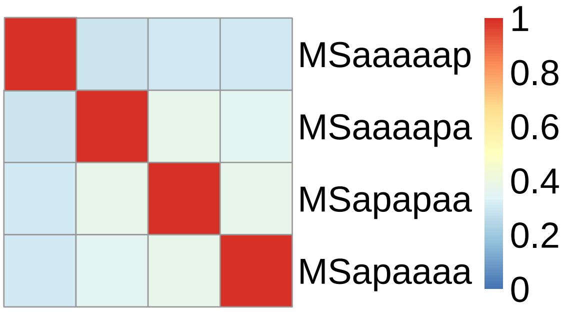

(b) Cell similarity heatmap

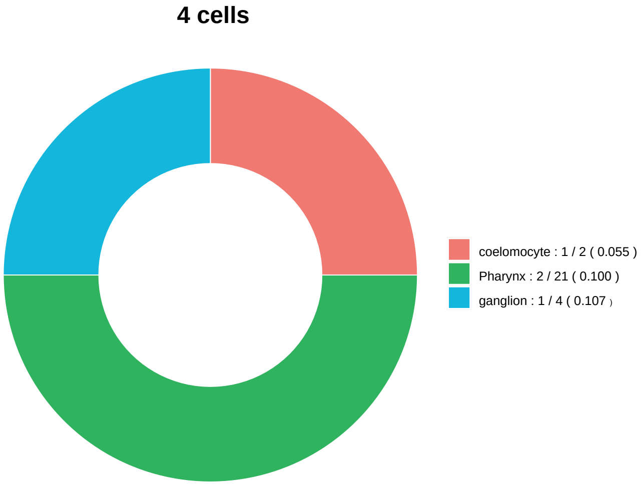

(c) Cell fate proportion plot

Figure S22: The third bicluster result obtained after removing the cell similarity component in the toy example. (a) Heatmap of Pearson correlation coefficient matrix between gene profiles. (b) Heatmap of KS test p-values matrix between cells. (c) Cell fate proportion diagram.

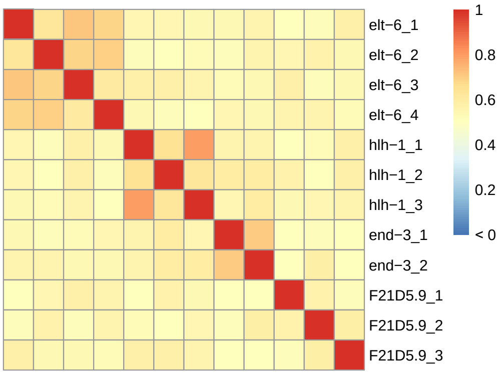

(a) Gene correlation heatmap

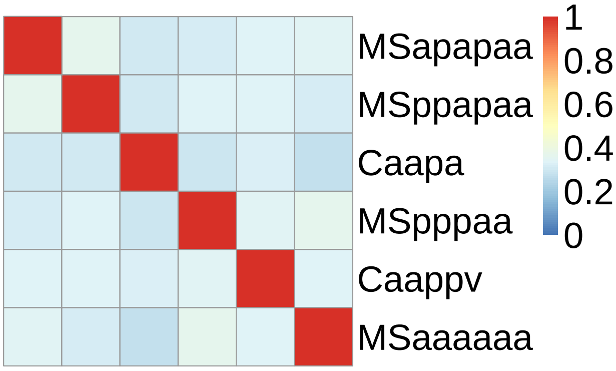

(b) Cell similarity heatmap

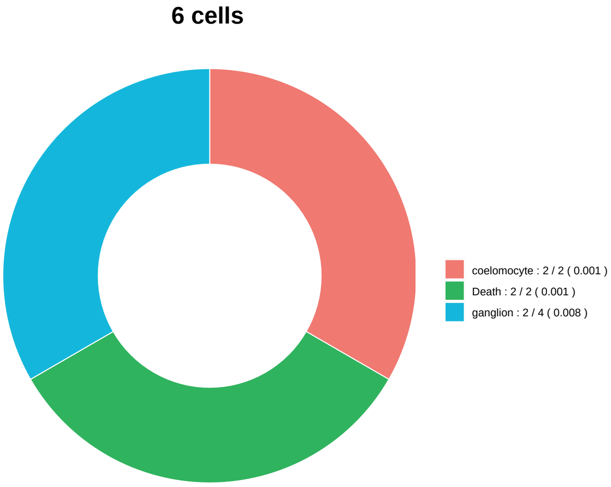

(c) Cell fate proportion plot

Figure S23: The fourth bicluster result obtained after removing the cell similarity component in the toy example. (a) Heatmap of Pearson correlation coefficient matrix between gene profiles. (b) Heatmap of KS test p-values matrix between cells. (c) Cell fate proportion diagram.

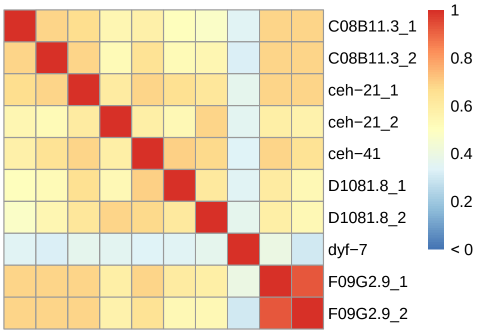

(a) Gene correlation heatmap

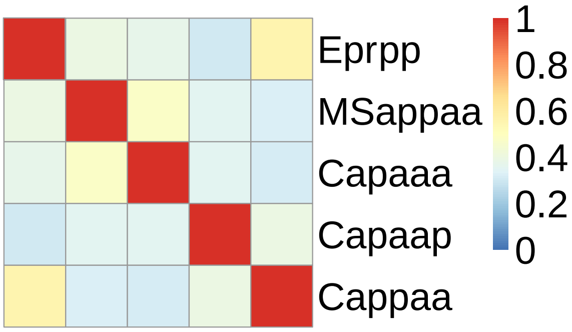

(b) Cell similarity heatmap

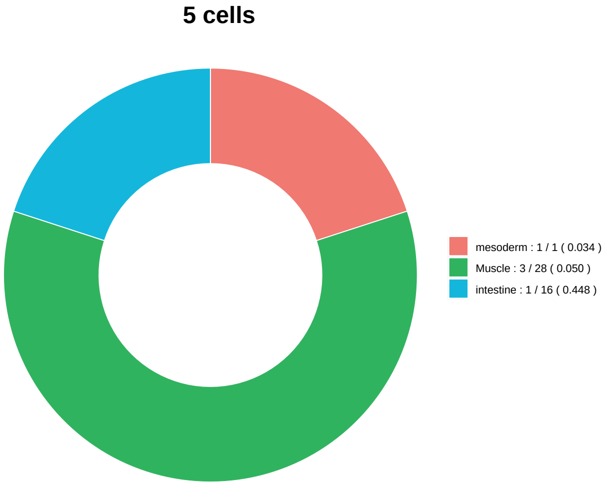

(c) Cell fate proportion plot

Figure S24: The fifth bicluster result obtained after removing the cell similarity component in the toy example. (a) Heatmap of Pearson correlation coefficient matrix between gene profiles. (b) Heatmap of KS test p-values matrix between cells. (c) Cell fate proportion diagram.

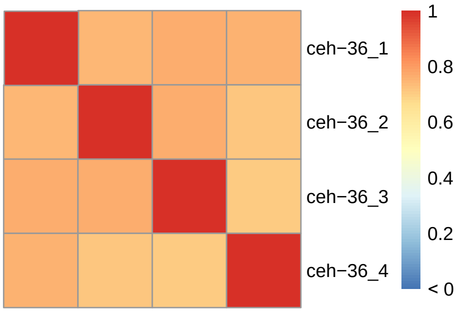

(a) Gene correlation heatmap

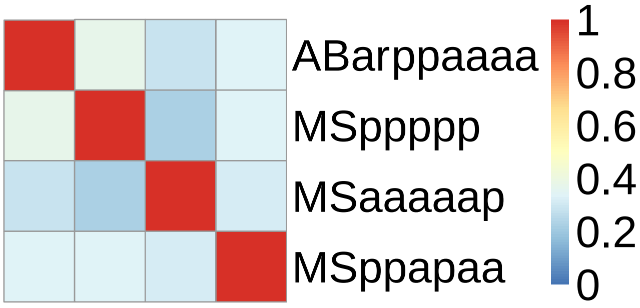

(b) Cell similarity heatmap

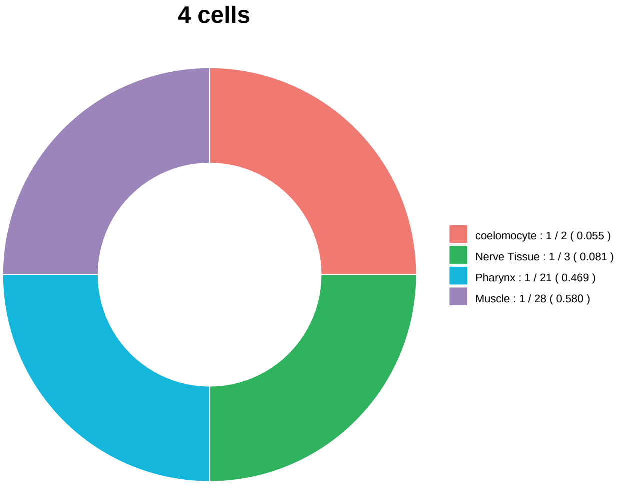

(c) Cell fate proportion plot

Figure S25: The sixth bicluster result obtained after removing the cell similarity component in the toy example. (a) Heatmap of Pearson correlation coefficient matrix between gene profiles. (b) Heatmap of KS test p-values matrix between cells. (c) Cell fate proportion diagram.

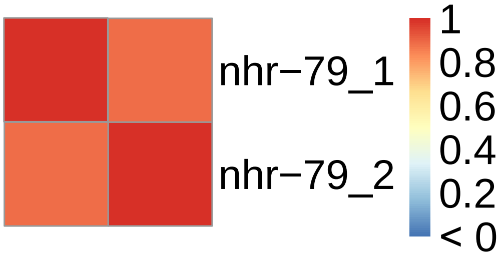

(a) Gene correlation heatmap

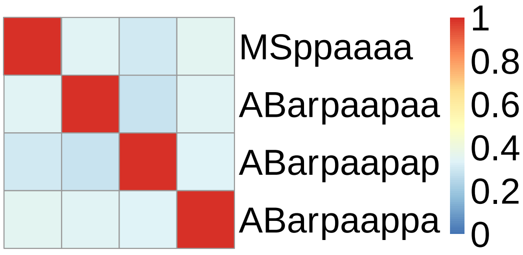

(b) Cell similarity heatmap

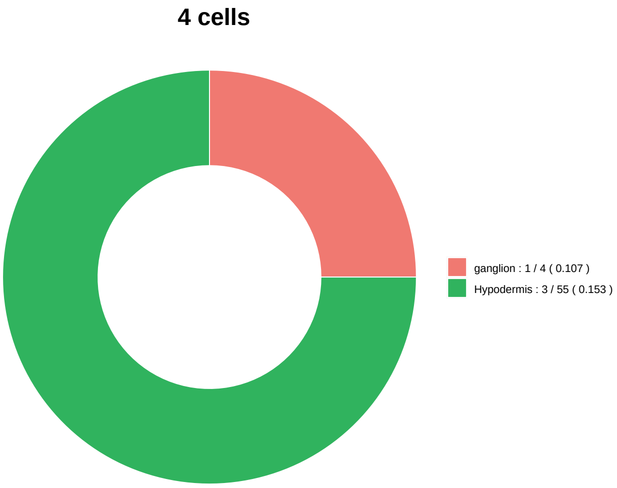

(c) Cell fate proportion plot

Figure S26: The seventh bicluster result obtained after removing the cell similarity component in the toy example. (a) Heatmap of Pearson correlation coefficient matrix between gene profiles. (b) Heatmap of KS test p-values matrix between cells. (c) Cell fate proportion diagram.

- The result after removing the gene expression size component:

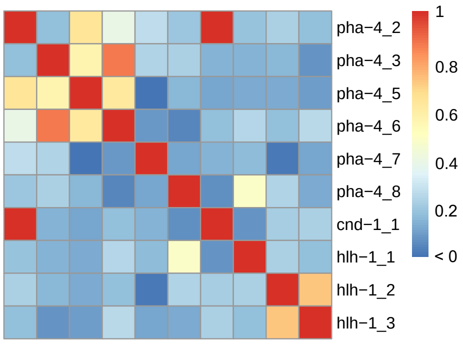

(a) Gene correlation heatmap

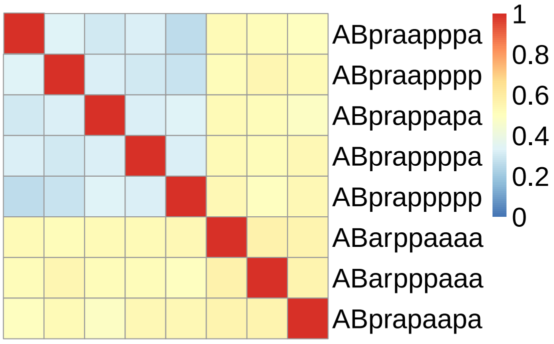

(b) Cell similarity heatmap

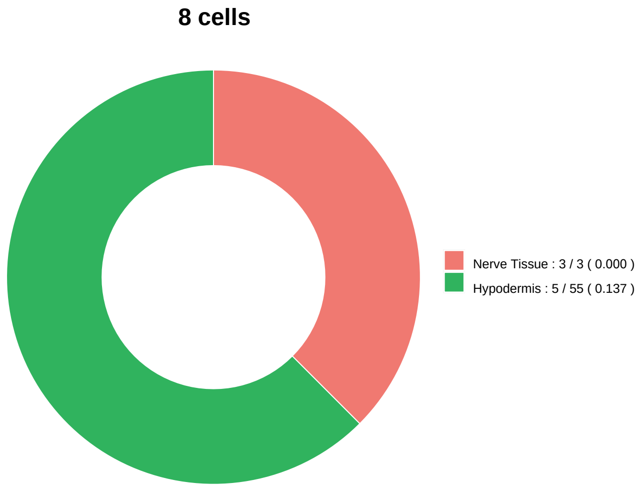

(c) Cell fate proportion plot

Figure S27: The first bicluster result obtained after removing the gene expression size component in the toy example. (a) Heatmap of Pearson correlation coefficient matrix between gene profiles. (b) Heatmap of KS test p-values matrix between cells. (c) Cell fate proportion diagram.

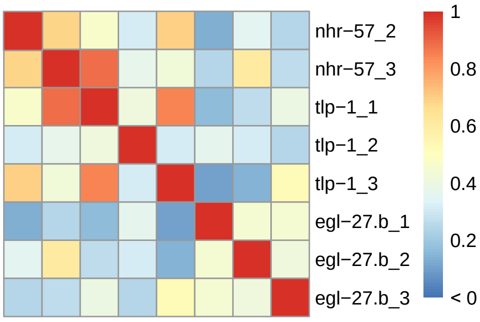

(a) Gene correlation heatmap

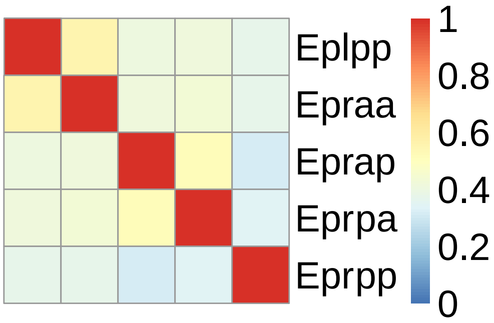

(b) Cell similarity heatmap

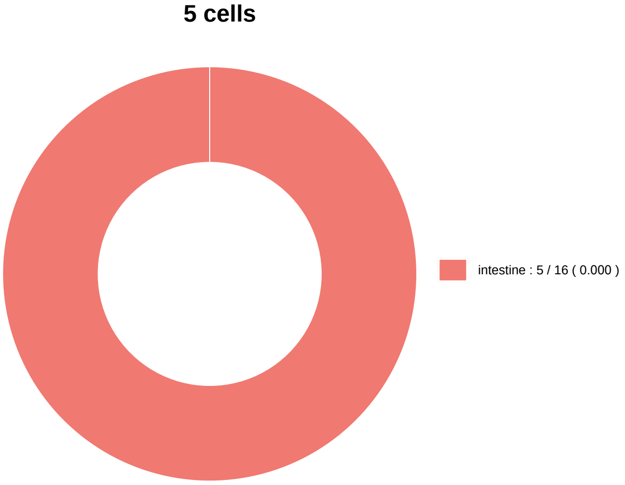

(c) Cell fate proportion plot

Figure S28: The second bicluster result obtained after removing the gene expression size component in the toy example. (a) Heatmap of Pearson correlation coefficient matrix between gene profiles. (b) Heatmap of KS test p-values matrix between cells. (c) Cell fate proportion diagram.

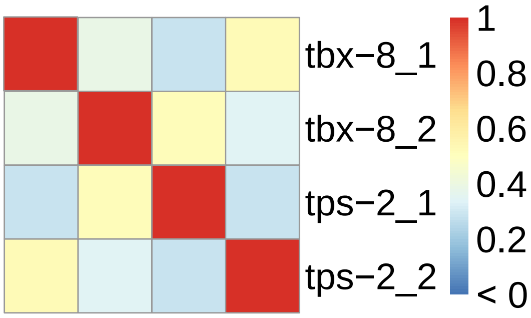

(a) Gene correlation heatmap

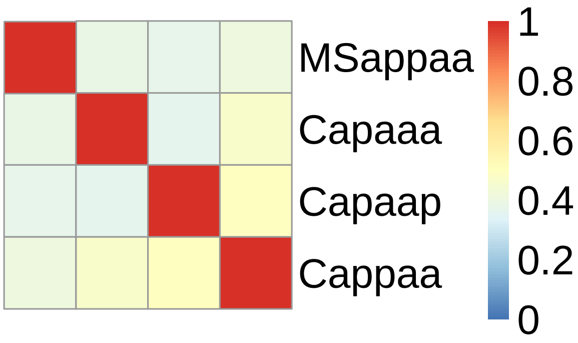

(b) Cell similarity heatmap

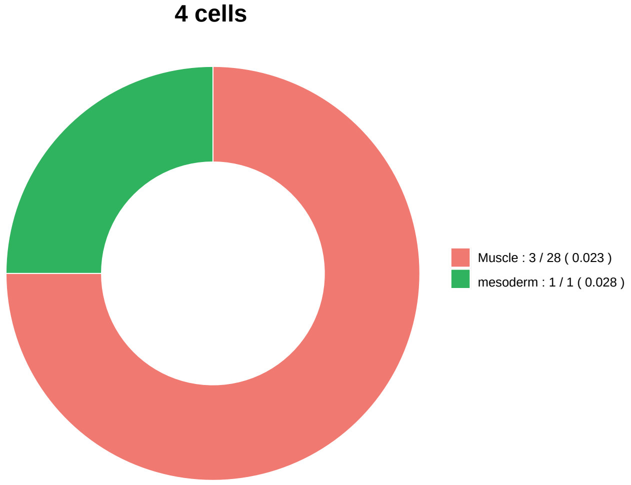

(c) Cell fate proportion plot

Figure S29: The third bicluster result obtained after removing the gene expression size component in the toy example. (a) Heatmap of Pearson correlation coefficient matrix between gene profiles. (b) Heatmap of KS test p-values matrix between cells. (c) Cell fate proportion diagram.

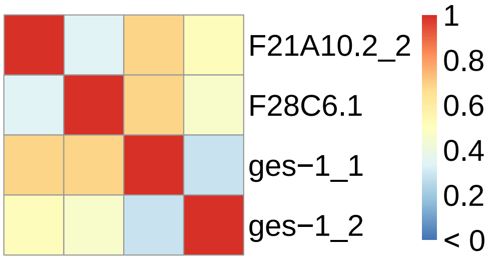

(a) Gene correlation heatmap

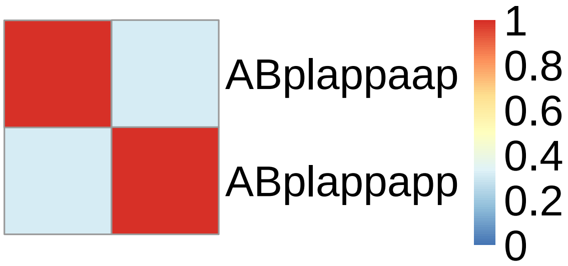

(b) Cell similarity heatmap

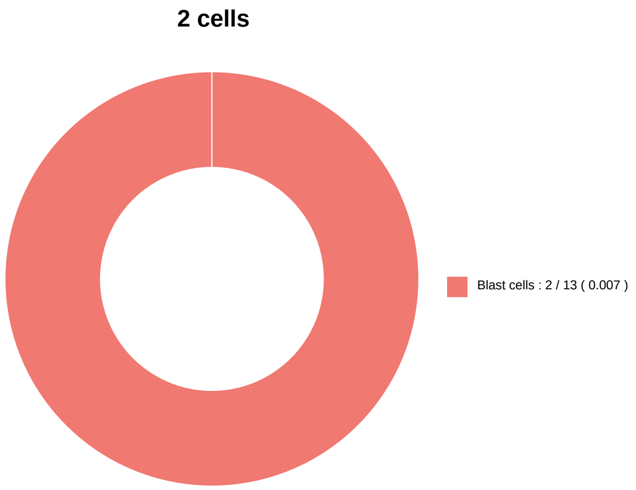

(c) Cell fate proportion plot

Figure S30: The fourth bicluster result obtained after removing the gene expression size component in the toy example. (a) Heatmap of Pearson correlation coefficient matrix between gene profiles. (b) Heatmap of KS test p-values matrix between cells. (c) Cell fate proportion diagram.

F Biclustering results on toy example

This dataset includes 51 copies from 13 different genes, and 145 cells with known cell fates, encompassing a total of 10 cell fates. The biclustering search algorithm is applied to the small-scale dataset, resulting in a total of nine biclusters. Heatmaps of gene expression and cell fate scale maps are generated for each bicluster. The first bicluster result has been presented in the manuscript, and the remaining bicluster results are as follows:

- The 2-nd biclustering result:

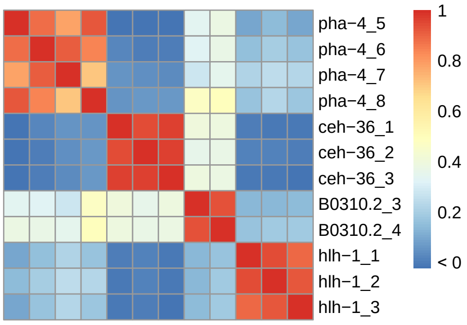

(a) Gene correlation heatmap

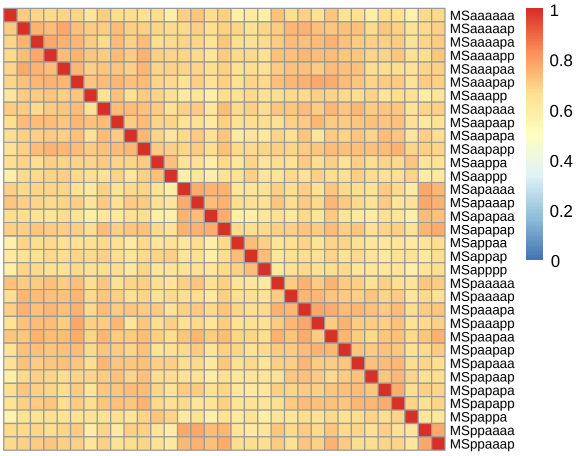

(b) Cell similarity heatmap

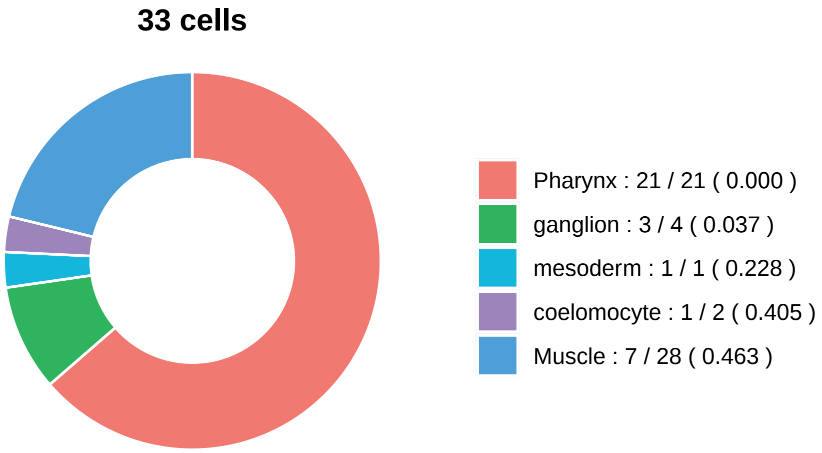

(c) Cell fate proportion plot

Figure S31: The second biclustering result on toy example. (a) Heatmap of Pearson correlation coefficient matrix between gene profiles. (b) Heatmap of KS test p-values matrix between cells. (c) Cell fate proportion diagram.

- The 3-rd biclustering result:

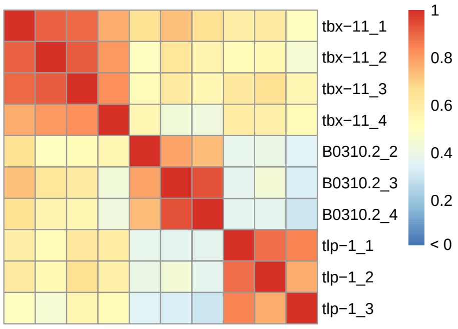

(a) Gene correlation heatmap

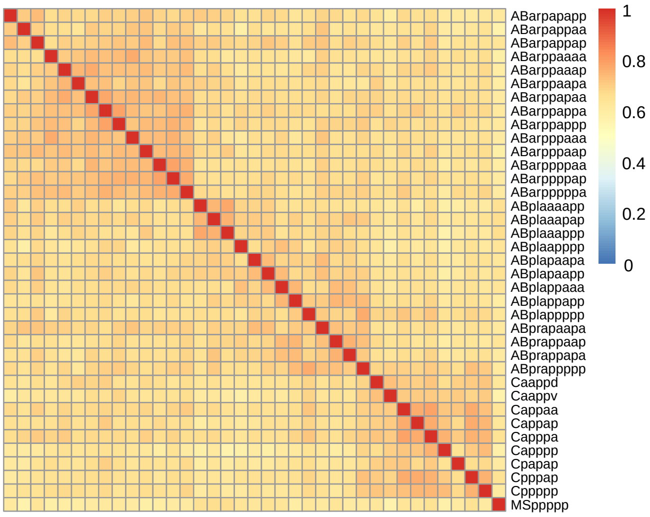

(b) Cell similarity heatmap

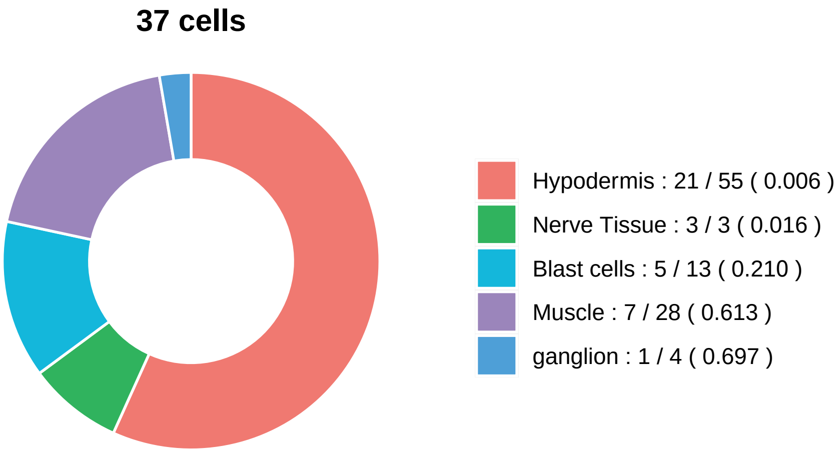

(c) Cell fate proportion plot

Figure S32: The third biclustering result on toy example. (a) Heatmap of Pearson correlation coefficient matrix between gene profiles. (b) Heatmap of KS test p-values matrix between cells. (c) Cell fate proportion diagram.

•The 4-th biclustering result

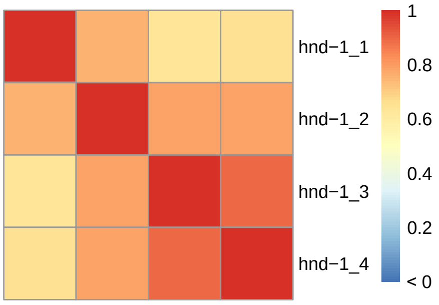

(a) Gene correlation heatmap

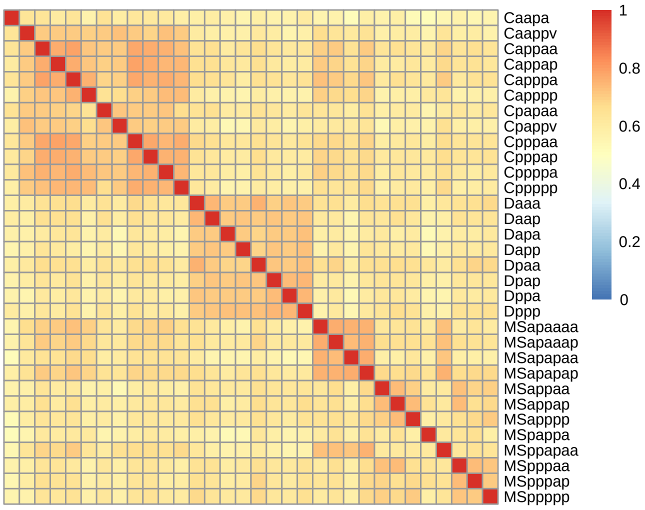

(b) Cell similarity heatmap

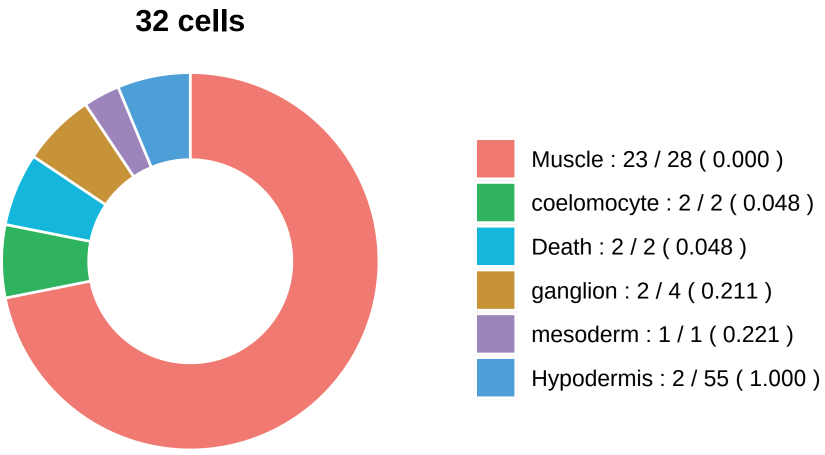

(c) Cell fate proportion plot

Figure S33: The fourth biclustering result on toy example. (a) Heatmap of Pearson correlation coefficient matrix between gene profiles. (b) Heatmap of KS test p-values matrix between cells. (c) Cell fate proportion diagram.

• The 5-th biclustering result

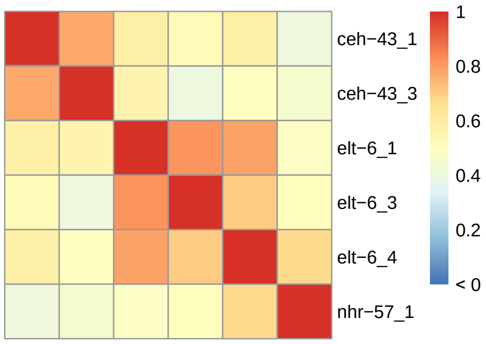

(a) Gene correlation heatmap

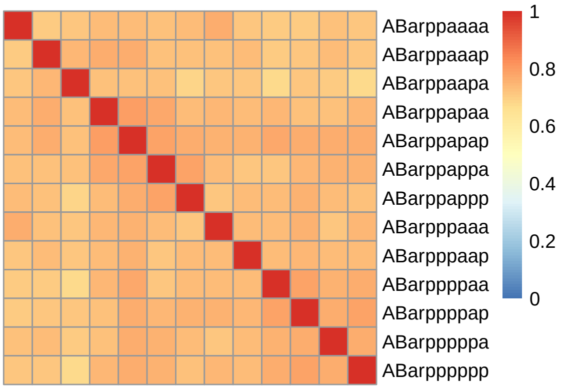

(b) Cell similarity heatmap

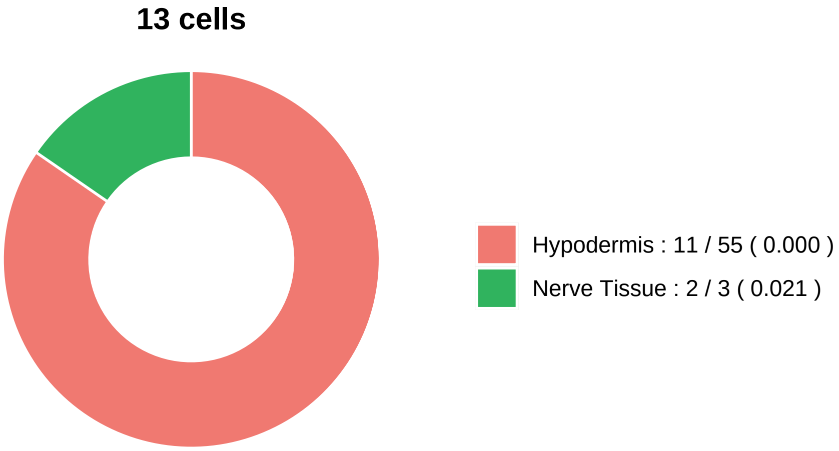

(c) Cell fate proportion plot

Figure S34: The fifth biclustering result on toy example. (a) Heatmap of Pearson correlation coefficient matrix between gene profiles. (b) Heatmap of KS test p-values matrix between cells. (c) Cell fate proportion diagram.

•The 6-th biclustering result

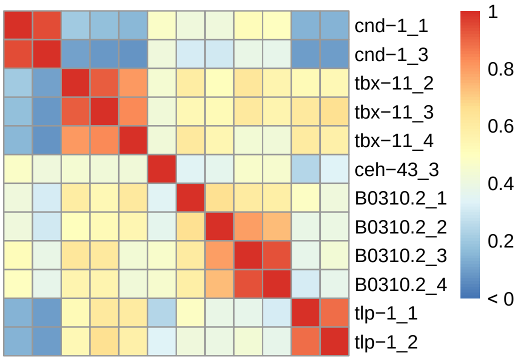

(a) Gene correlation heatmap

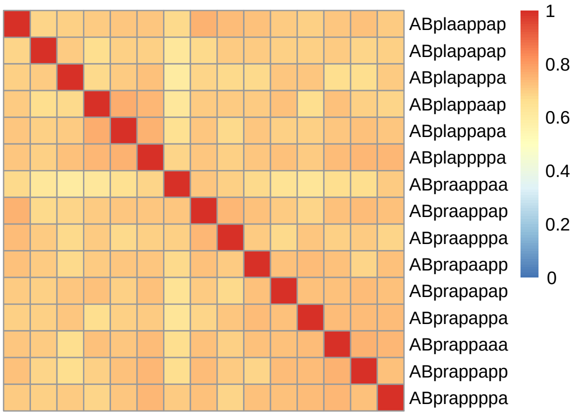

(b) Cell similarity heatmap

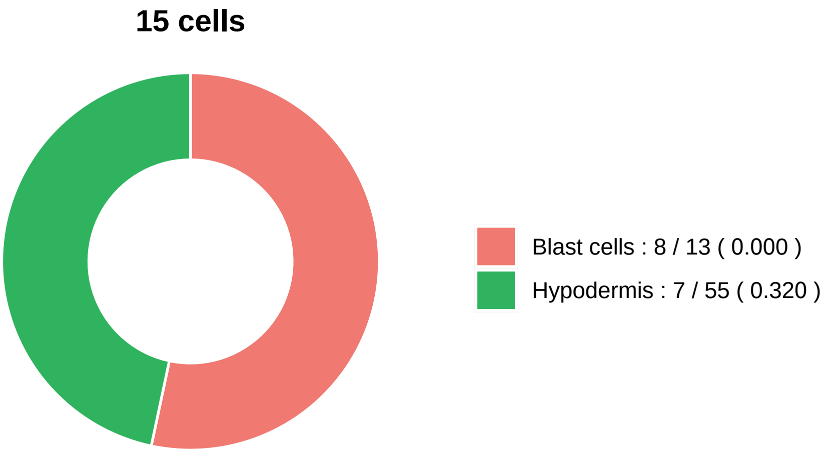

(c) Cell fate proportion plot

Figure S35: The sixth biclustering result on toy example. (a) Heatmap of Pearson correlation coefficient matrix between gene profiles. (b) Heatmap of KS test p-values matrix between cells. (c) Cell fate proportion diagram.

•The 7-th biclustering result

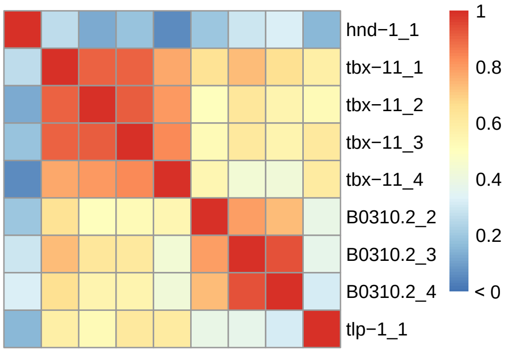

(a) Gene correlation heatmap

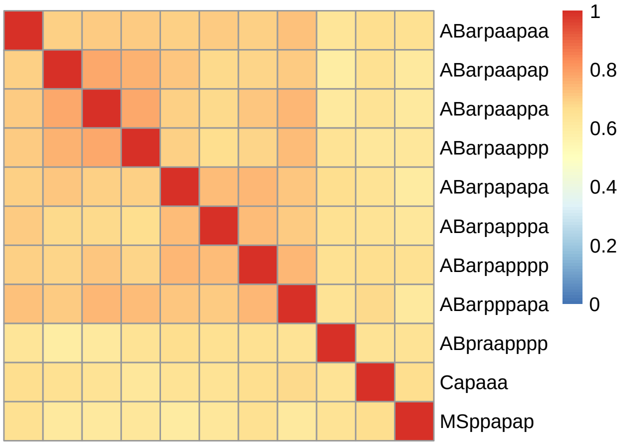

(b) Cell similarity heatmap

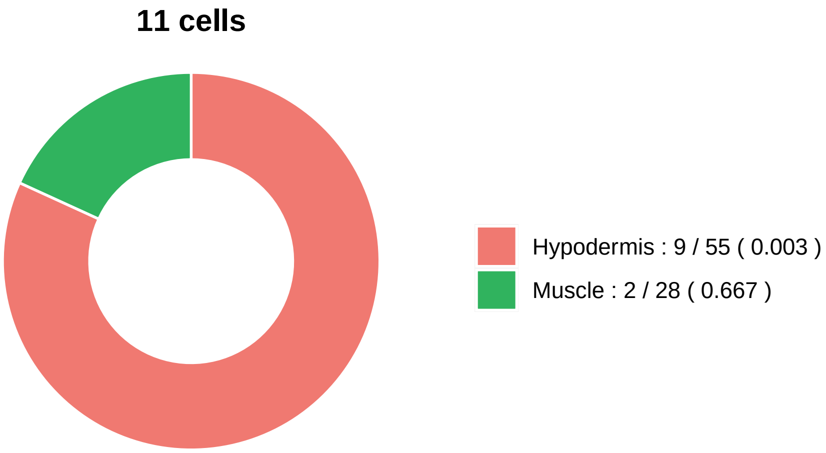

(c) Cell fate proportion plot

Figure S36: The seventh biclustering result on toy example. (a) Heatmap of Pearson correlation coefficient matrix between gene profiles. (b) Heatmap of KS test p-values matrix between cells. (c) Cell fate proportion diagram.

•The 8-th biclustering result

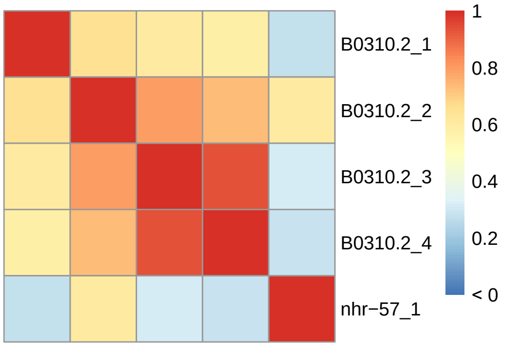

(a) Gene correlation heatmap

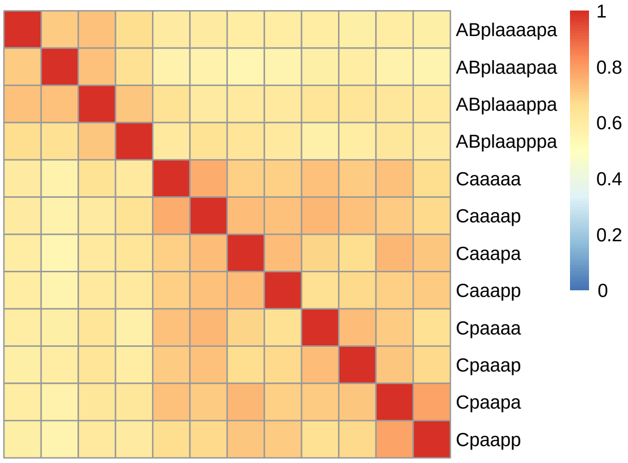

(b) Cell similarity heatmap

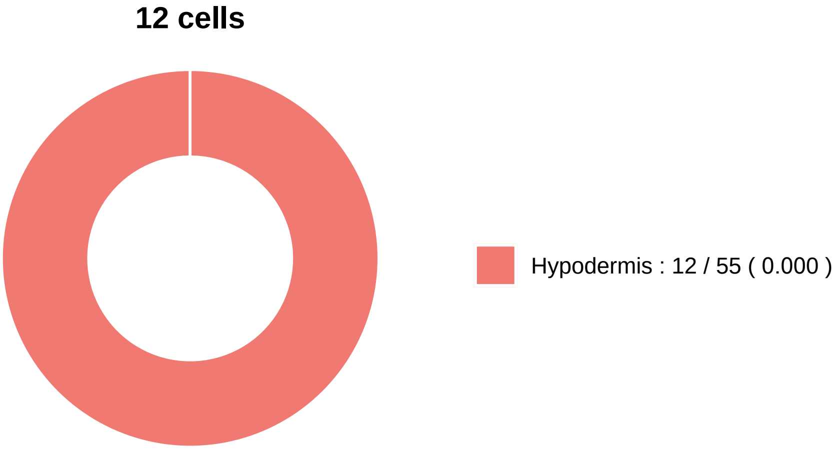

(c) Cell fate proportion plot

Figure S37: The eighth biclustering result on toy example. (a) Heatmap of Pearson correlation coefficient matrix between gene profiles. (b) Heatmap of KS test p-values matrix between cells. (c) Cell fate proportion diagram.

•The 9-th biclustering result

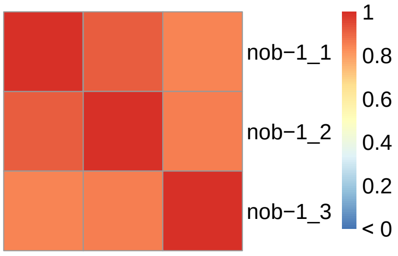

(a) Gene correlation heatmap

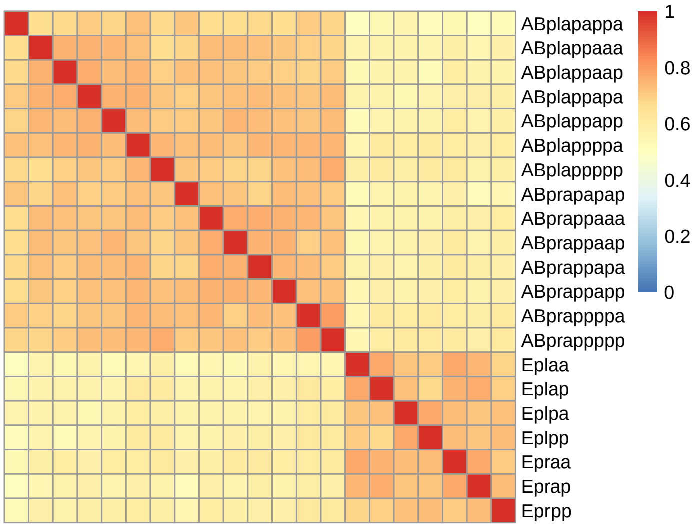

(b) Cell similarity heatmap

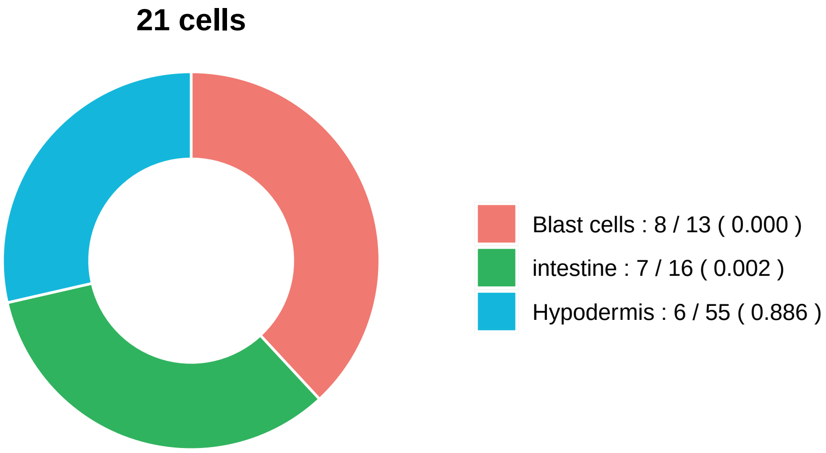

(c) Cell fate proportion plot

Figure S38: The ninth biclustering result on toy example. (a) Heatmap of Pearson correlation coefficient matrix between gene profiles. (b) Heatmap of KS test p-values matrix between cells. (c) Cell fate proportion diagram.

G Comparison with other biclustering models

Five comparative biclustering models, including the CC model proposed in Cheng and Church (2000), the plaid model proposed in Lazzeroni and Owen (2002), the xMOTIFs model proposed in Murali and Kasif (2002), the QUBIC2 model proposed in Xie et al. (2020), and the ARBic model proposed in (Liu et al., 2023), are applied to the toy example dataset. The computational methods for the five models are as follows:

• CC Model: The CC model employs an iterative clustering-based approach. It first divides the dataset into multiple subsets and then identifies highly similar rows and columns by performing clustering on these subsets. This process iterates until finding bicluster patterns that meet specific criteria.

• Plaid Model: The Plaid model adopts Boolean factorization. It represents the dataset as the product of two binary matrices, each corresponding to the rows and columns of the dataset. Then, by adjusting these two matrices to minimize the reconstruction error of the dataset, the Plaid model discovers bicluster patterns within the dataset.

• xMOTIFs: The xMOTIFs model utilizes a method based on pattern repeatability to discover bicluster patterns. It identifies frequently occurring bidirectional patterns in the dataset by traversing patterns of different lengths, thus uncovering the most significant biclusters.

• QUBIC2: The QUBIC2 model combines biclustering and factorization. It discovers bicluster patterns within the dataset by minimizing the reconstruction error while considering clustering information for both rows and columns.

• ARBic: The ARBic model is based on model selection criteria, such as BIC. It partitions the data based on biclustering and aims to find the optimal bicluster patterns. It seeks to maximize the adaptiveness of the model while minimizing its complexity.

Multiple experiments are conducted on these biclustering models, but they fail to produce satisfactory biclustering outcomes. The CC model and xMOTIFs model do not identify any biclusters, while the plaid model, QUBIC2 model, and ARBic model yield extremely poor results. In these results, genes within clusters are not expressed in the corresponding cells, and there are no meaningful patterns or relationships between genes and cells. The plaid model, QUBIC2 model, and ARBic model are all used with parameter settings recommended in their respective literature, and the best results are shown in the following figures, revealing three, four, and five biclusters, respectively.

•The plaid model

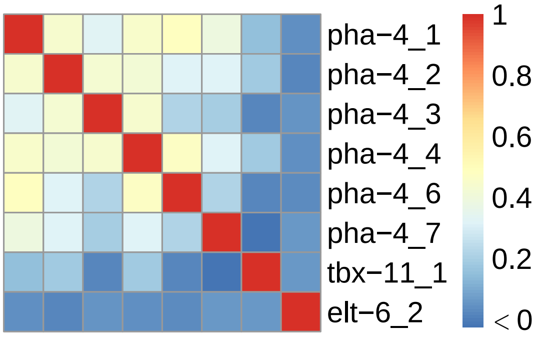

(a) Gene correlation heatmap

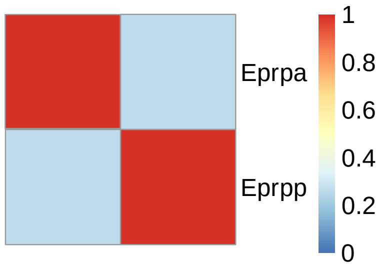

(b) Cell similarity heatmap

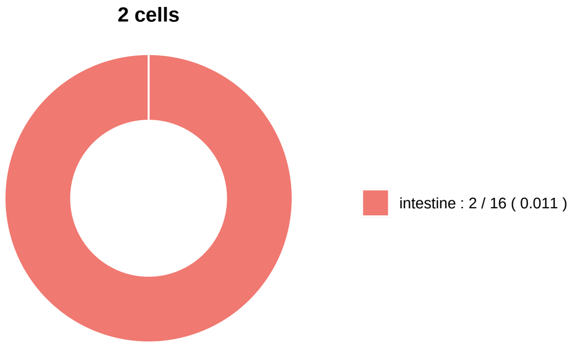

(c) Cell fate proportion plot

Figure S39: The first biclustering result of the plaid model on toy example. (a) Heatmap of Pearson correlation coefficient matrix between gene profiles. (b) Heatmap of KS test p-values matrix between cells. (c) Cell fate proportion diagram.

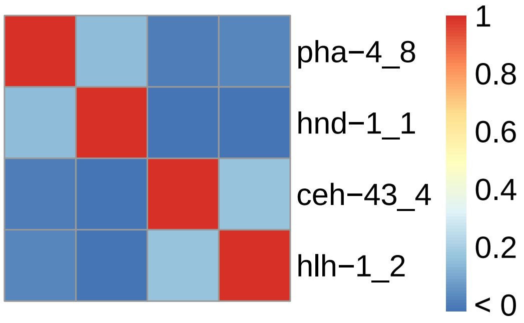

(a) Gene correlation heatmap

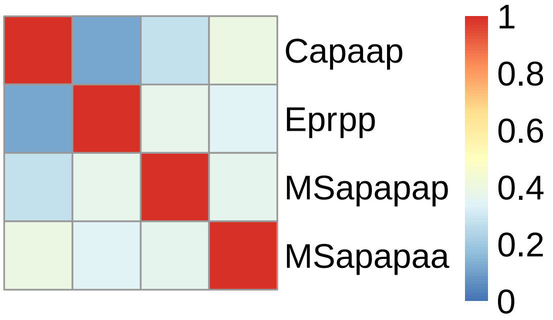

(b) Cell similarity heatmap

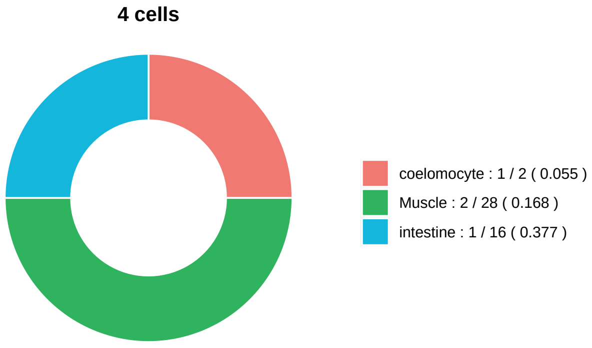

(c) Cell fate proportion plot

Figure S40: The second biclustering result of the plaid model on toy example. (a) Heatmap of Pearson correlation coefficient matrix between gene profiles. (b) Heatmap of KS test p-values matrix between cells. (c) Cell fate proportion diagram.

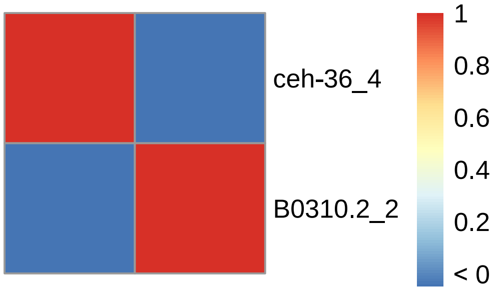

(a) Gene correlation heatmap

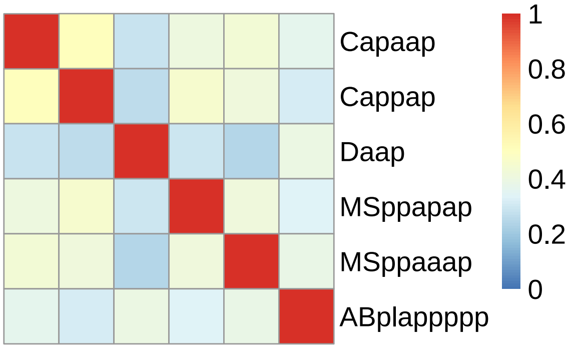

(b) Cell similarity heatmap

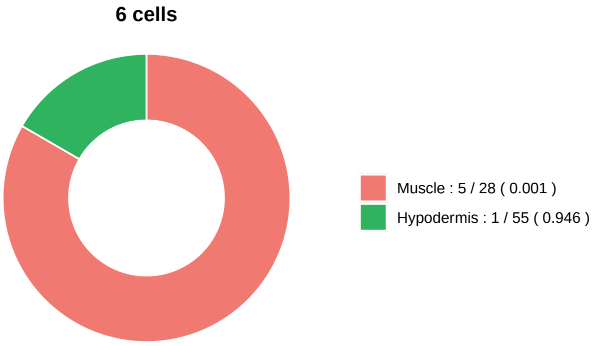

(c) Cell fate proportion plot

Figure S41: The third biclustering result of the plaid model on toy example. (a) Heatmap of Pearson correlation coefficient matrix between gene profiles. (b) Heatmap of KS test p-values matrix between cells. (c) Cell fate proportion diagram.

•The QUBIC2 model

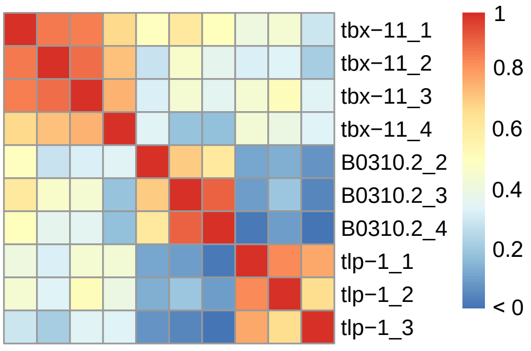

(a) Gene correlation heatmap

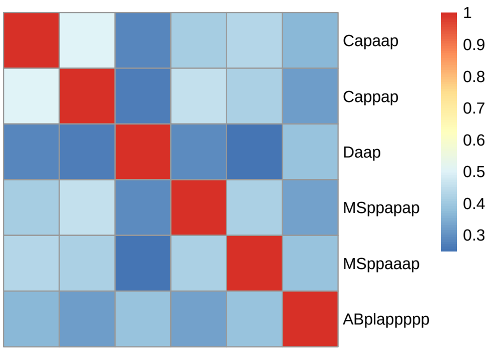

(b) Cell similarity heatmap

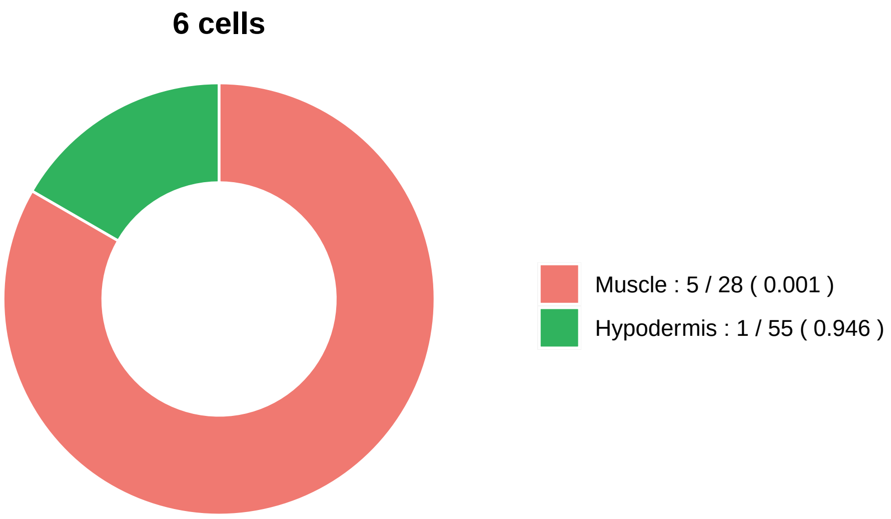

(c) Cell fate proportion plot

Figure S42: The first biclustering result of the QUBIC2 model on toy example. (a) Heatmap of Pearson correlation coefficient matrix between gene profiles. (b) Heatmap of KS test p-values matrix between cells. (c) Cell fate proportion diagram.

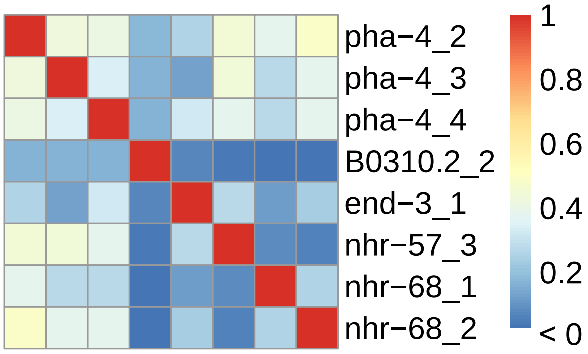

(a) Gene correlation heatmap

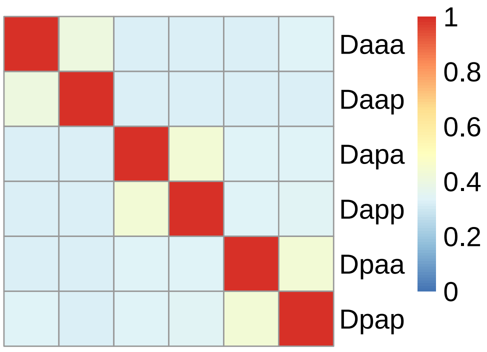

(b) Cell similarity heatmap

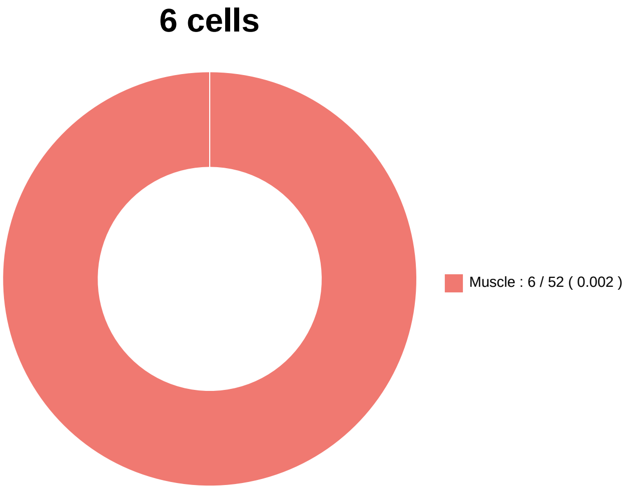

(c) Cell fate proportion plot

Figure S43: The second biclustering result of the QUBIC2 model on toy example. (a) Heatmap of Pearson correlation coefficient matrix between gene profiles. (b) Heatmap of KS test p-values matrix between cells. (c) Cell fate proportion diagram.

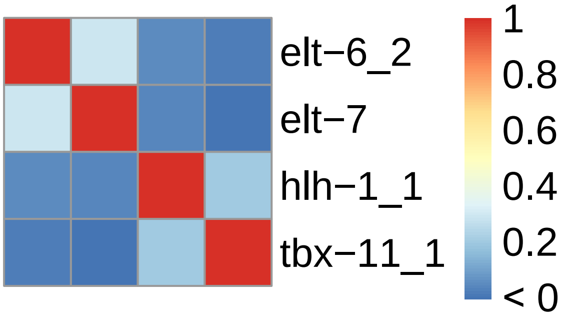

(a) Gene correlation heatmap

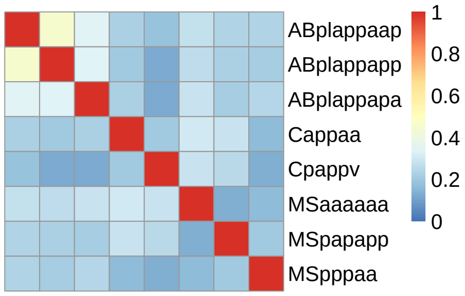

(b) Cell similarity heatmap

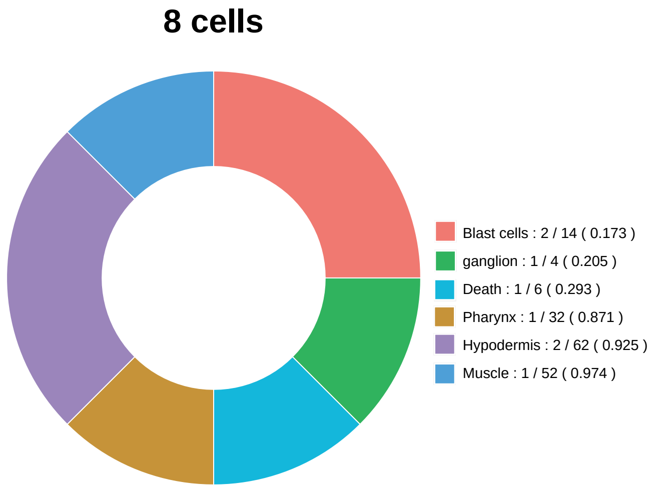

(c) Cell fate proportion plot

Figure S44: The third biclustering result of the QUBIC2 model on toy example. (a) Heatmap of Pearson correlation coefficient matrix between gene profiles. (b) Heatmap of KS test p-values matrix between cells. (c) Cell fate proportion diagram.

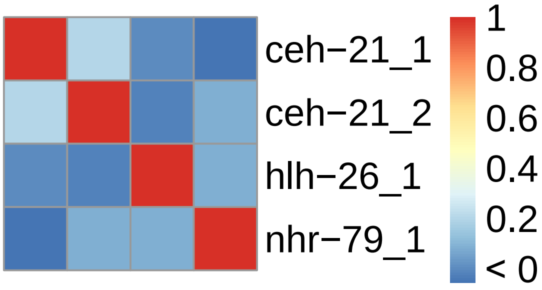

(a) Gene correlation heatmap

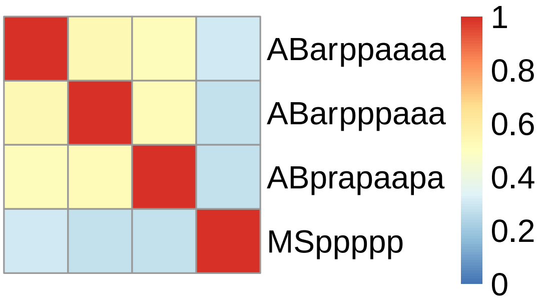

(b) Cell similarity heatmap

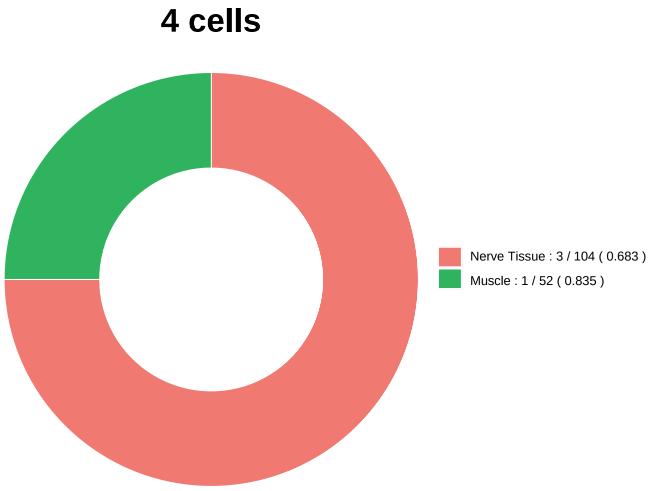

(c) Cell fate proportion plot

Figure S45: The fourth biclustering result of the QUBIC2 model on toy example. (a) Heatmap of Pearson correlation coefficient matrix between gene profiles. (b) Heatmap of KS test p-values matrix between cells. (c) Cell fate proportion diagram.

•The ARBic model

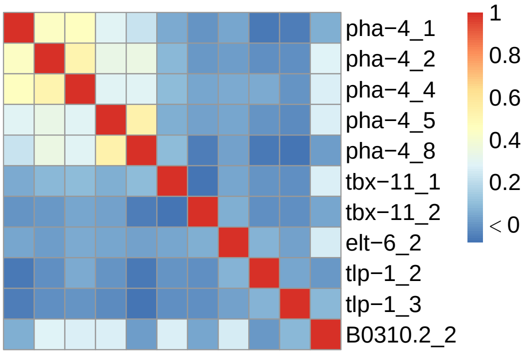

(a) Gene correlation heatmap

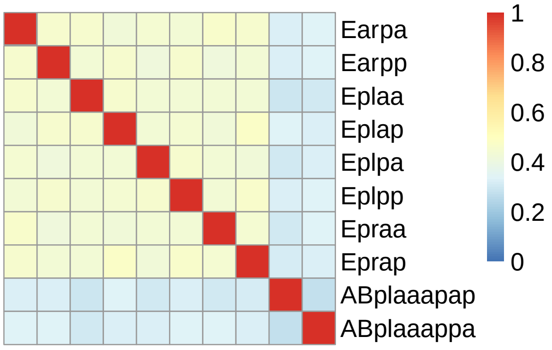

(b) Cell similarity heatmap

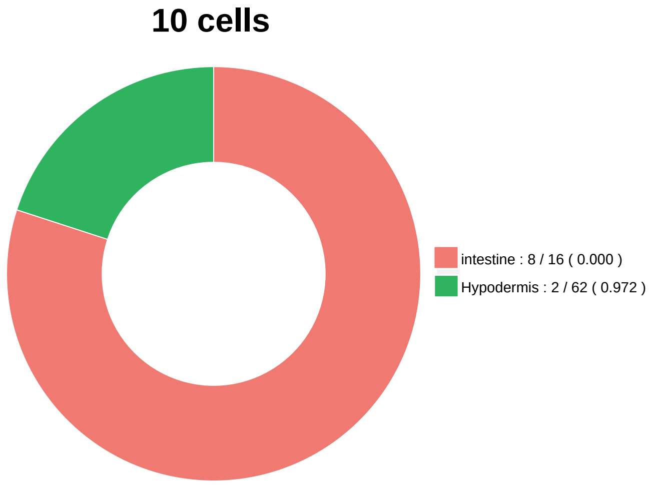

(c) Cell fate proportion plot

Figure S46: The first biclustering result of the ARBic model on toy example. (a) Heatmap of Pearson correlation coefficient matrix between gene profiles. (b) Heatmap of KS test p-values matrix between cells. (c) Cell fate proportion diagram.

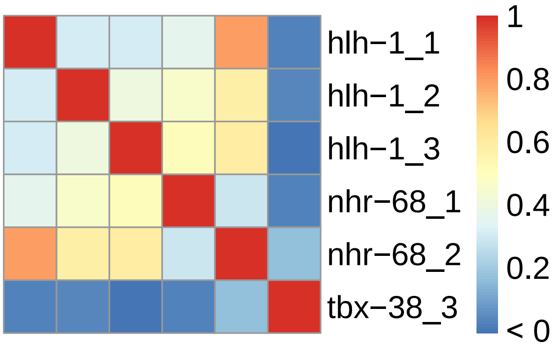

(a) Gene correlation heatmap

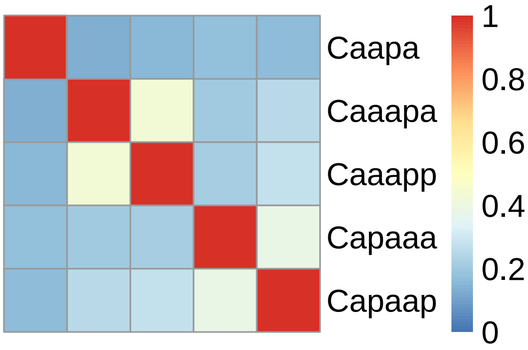

(b) Cell similarity heatmap

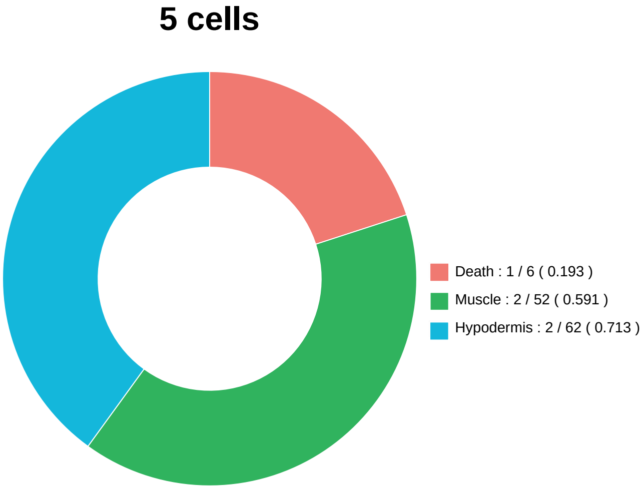

(c) Cell fate proportion plot

Figure S47: The second biclustering result of the ARBic model on toy example. (a) Heatmap of Pearson correlation coefficient matrix between gene profiles. (b) Heatmap of KS test p-values matrix between cells. (c) Cell fate proportion diagram.

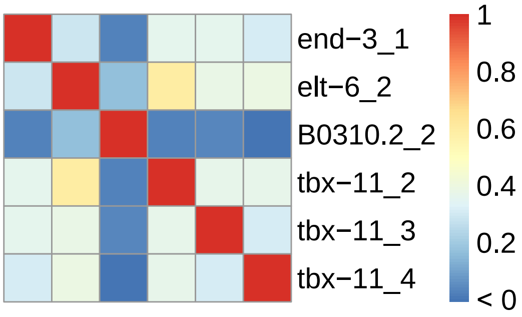

(a) Gene correlation heatmap

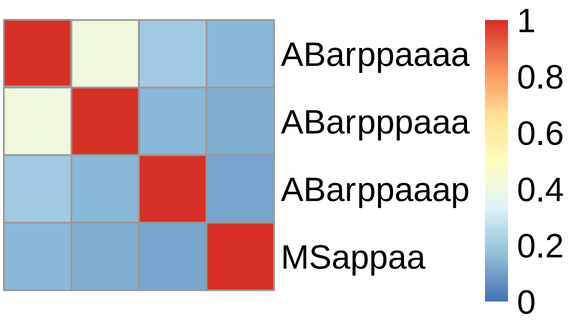

(b) Cell similarity heatmap

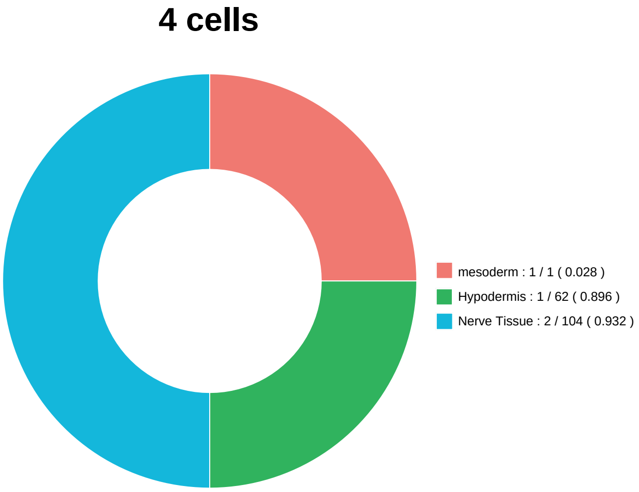

(c) Cell fate proportion plot

Figure S48: The third biclustering result of the ARBic model on toy example. (a) Heatmap of Pearson correlation coefficient matrix between gene profiles. (b) Heatmap of KS test p-values matrix between cells. (c) Cell fate proportion diagram.

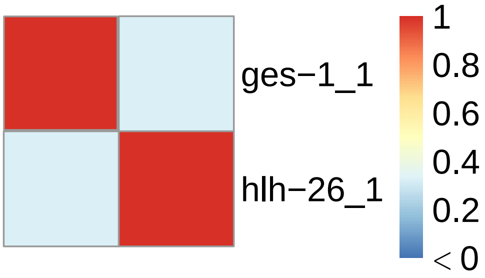

(a) Gene correlation heatmap

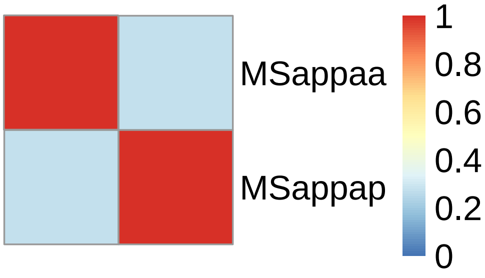

(b) Cell similarity heatmap

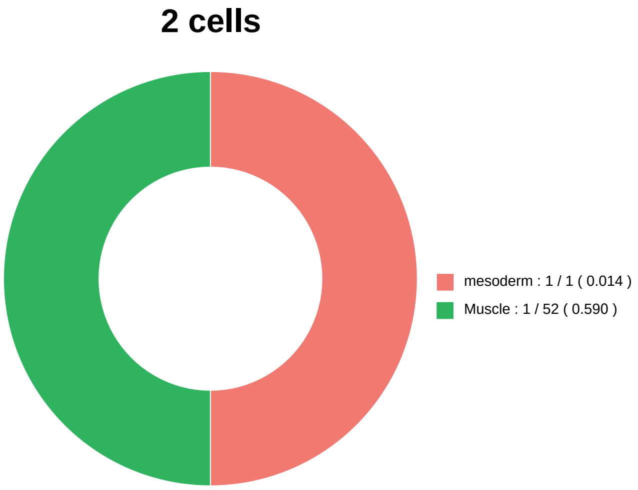

(c) Cell fate proportion plot

Figure S49: The fourth biclustering result of the ARBic model on toy example. (a) Heatmap of Pearson correlation coefficient matrix between gene profiles. (b) Heatmap of KS test p-values matrix between cells. (c) Cell fate proportion diagram.

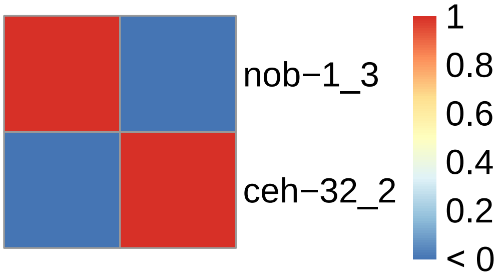

(a) Gene correlation heatmap

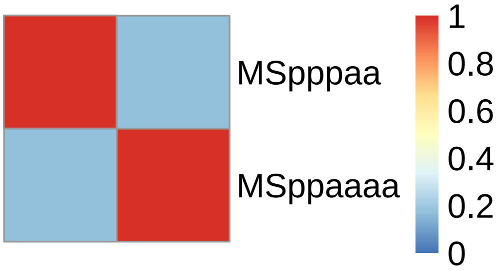

(b) Cell similarity heatmap

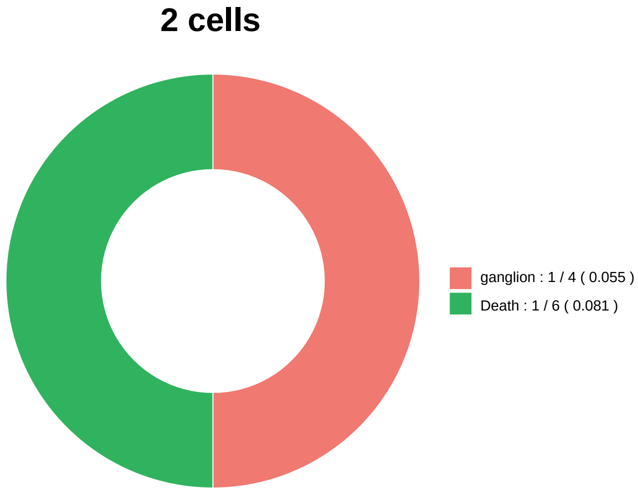

(c) Cell fate proportion plot

Figure S50: The fifth biclustering result of the ARBic model on toy example. (a) Heatmap of Pearson correlation coefficient matrix between gene profiles. (b) Heatmap of KS test p-values matrix between cells. (c) Cell fate proportion diagram.

H Biclustering results on the complete real data

Our complete dataset consists of 174 copies (corresponding to 104 different genes) and 724 cells (including 145 cells with known cell fates). The biclustering search algorithm is applied to the complete dataset of *C.elegans*, yielding a total of ten biclusters. The first bicluster result has been presented in the manuscript, and the remaining bicluster results are as follows:

•The 2-nd biclustering result

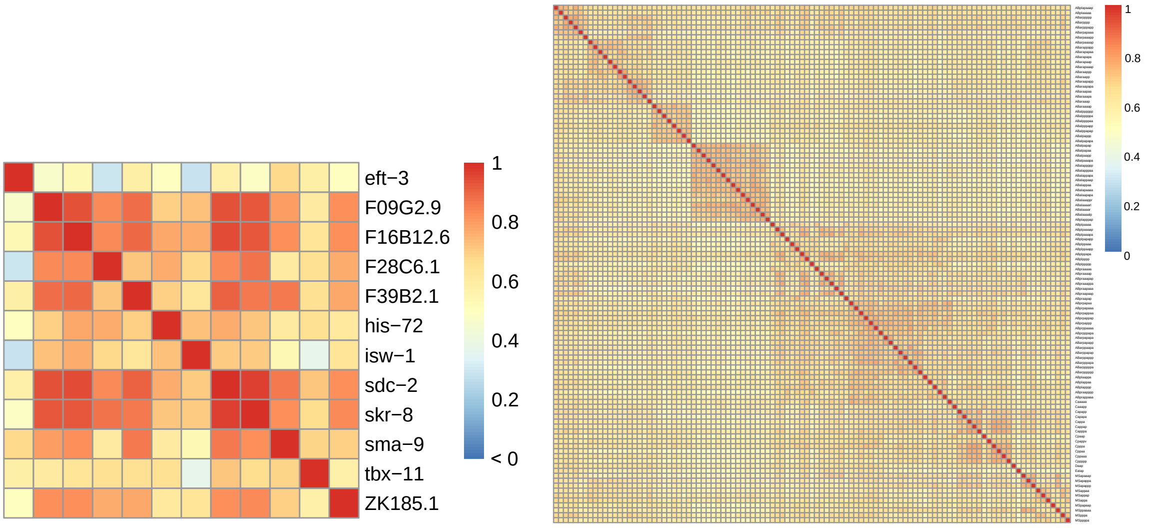

Figure S51: Gene expression heatmap and cell similarity heatmap

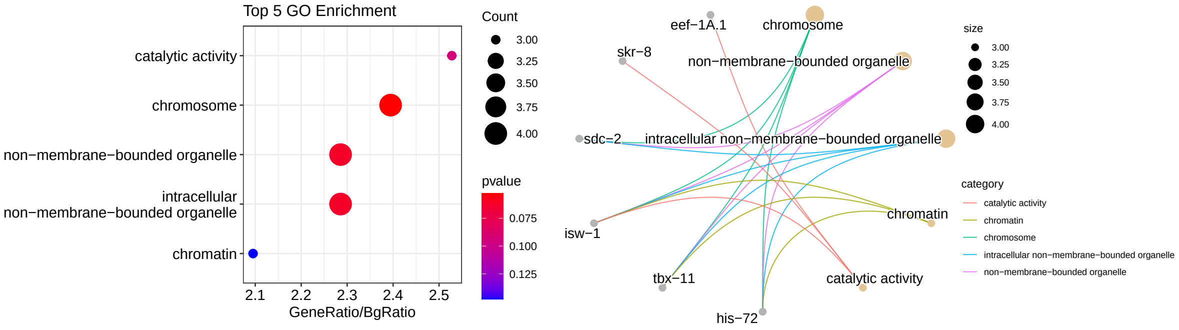

Figure S52: Gene enrichment analysis bubble plot and gene network diagrams for each pathway

•The 3-rd biclustering result

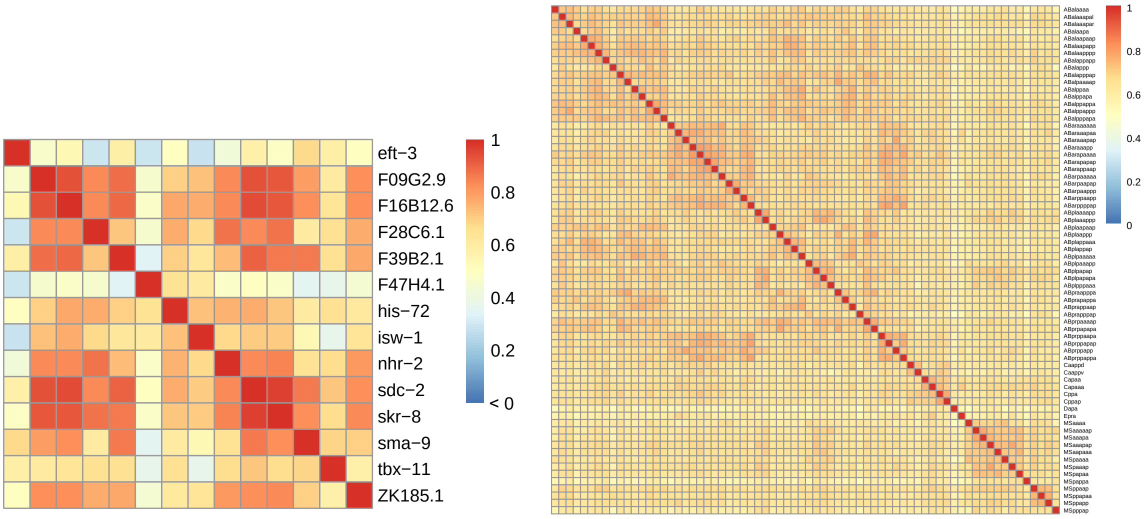

Figure S53: Gene expression heatmap and cell similarity heatmap

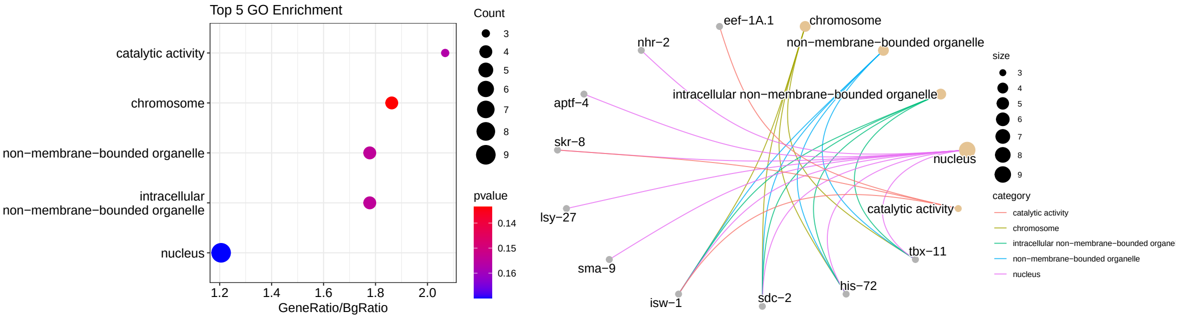

Figure S54: Gene enrichment analysis bubble plot and gene network diagrams for each pathway

•The 4-th biclustering result

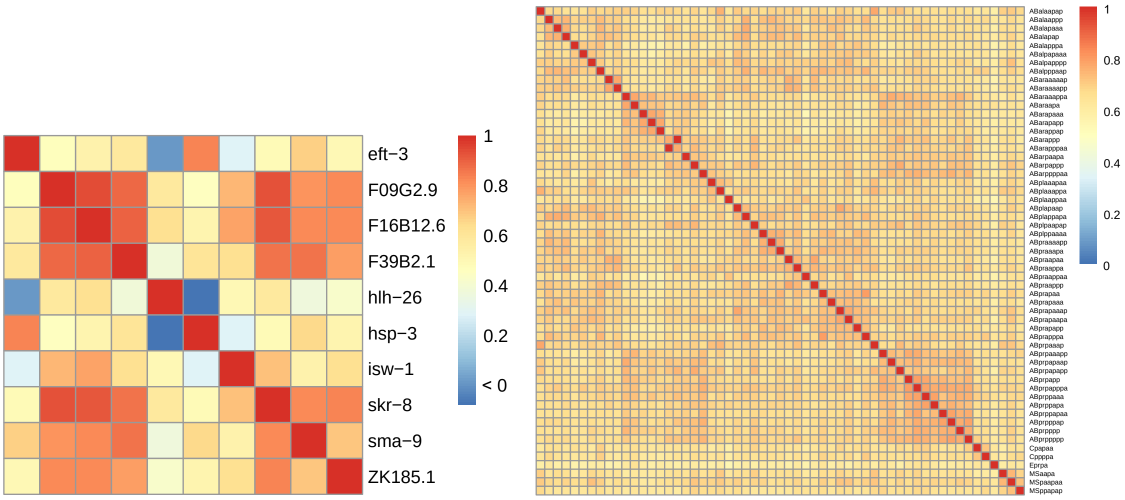

Figure S55: Gene expression heatmap and cell similarity heatmap

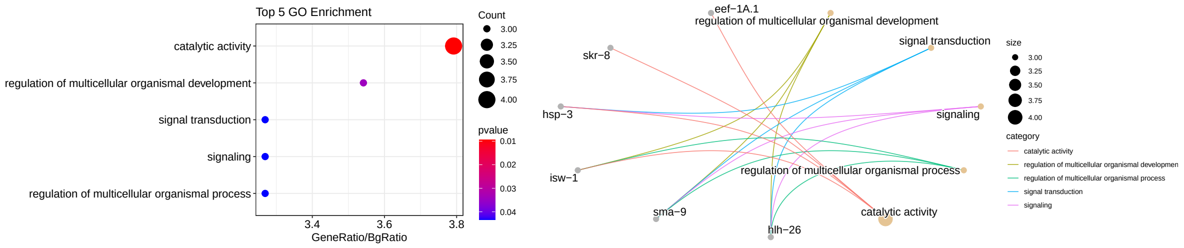

Figure S56: Gene enrichment analysis bubble plot and gene network diagrams for each pathway

•The 5-th biclustering result

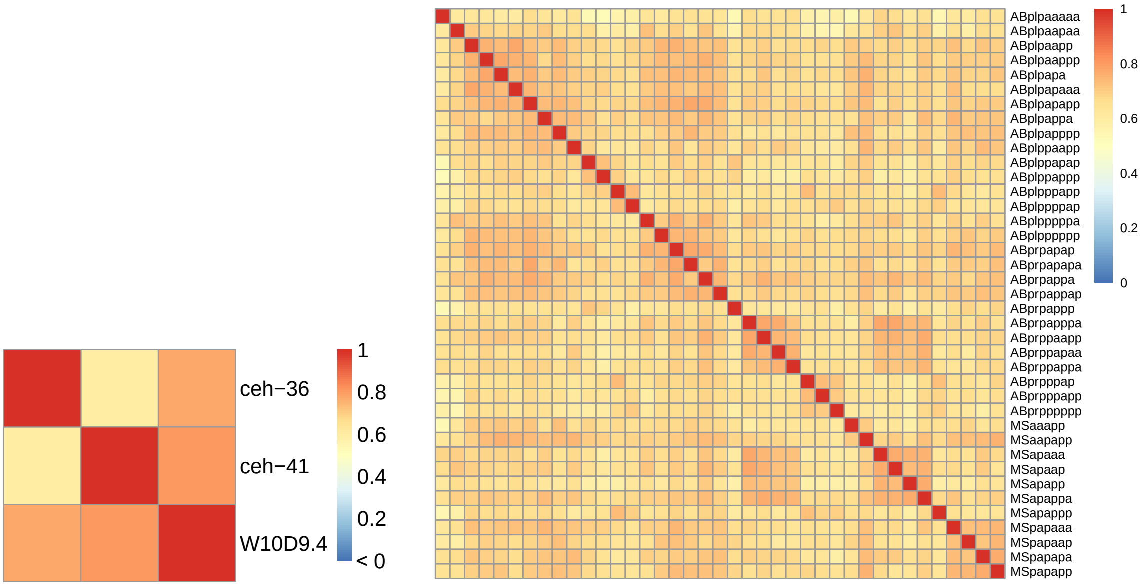

Figure S57: Gene expression heatmap and cell similarity heatmap

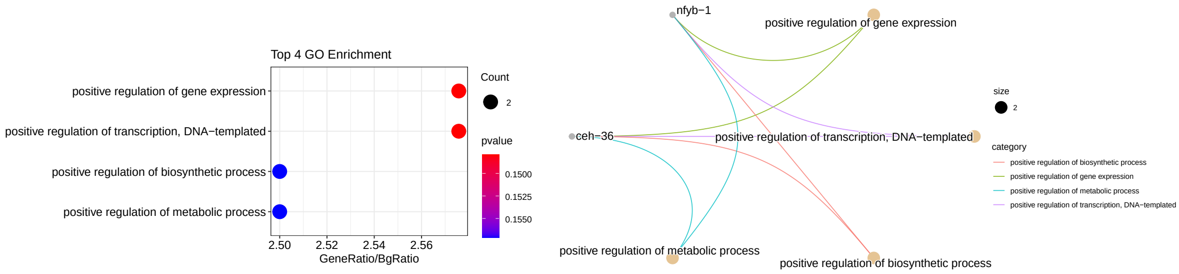

Figure S58: Gene enrichment analysis bubble plot and gene network diagrams for each pathway

•The 6-th biclustering result

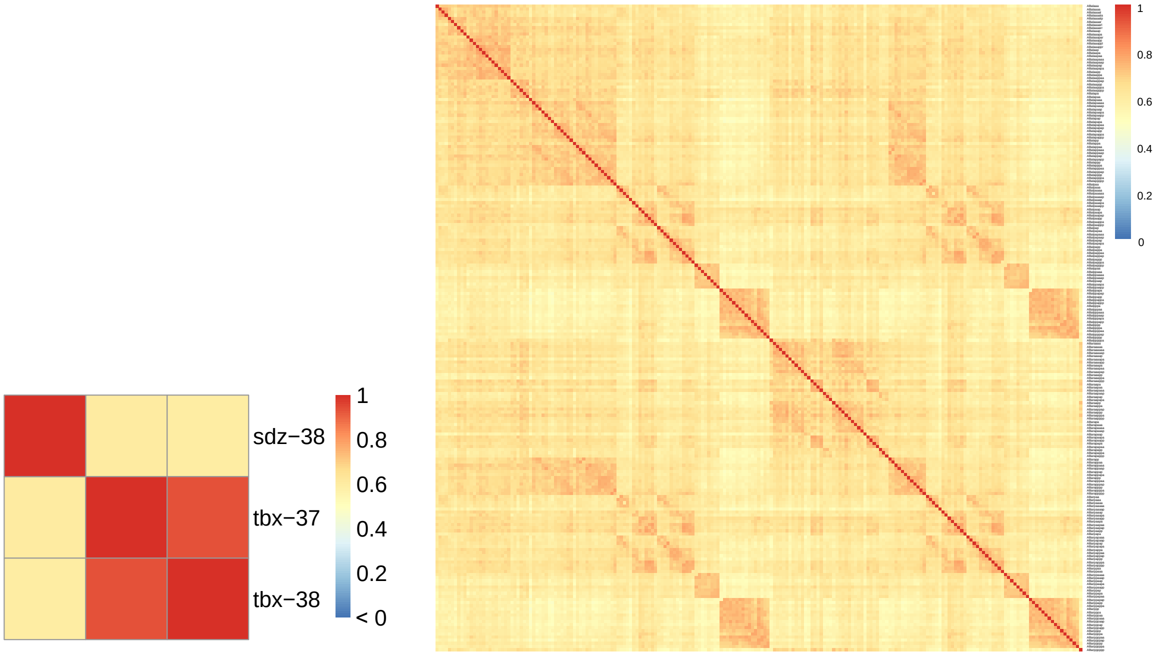

Figure S59: Gene expression heatmap and cell similarity heatmap

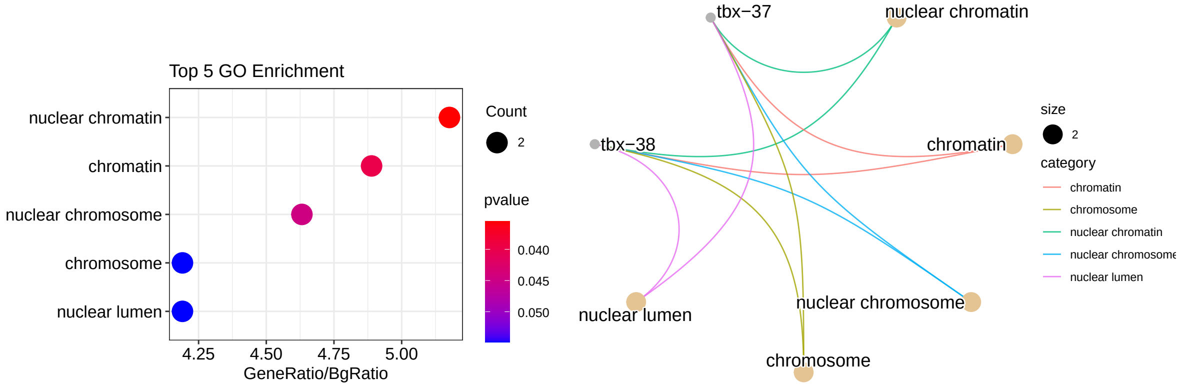

Figure S60: Gene enrichment analysis bubble plot and gene network diagrams for each pathway

•The 7-th biclustering result

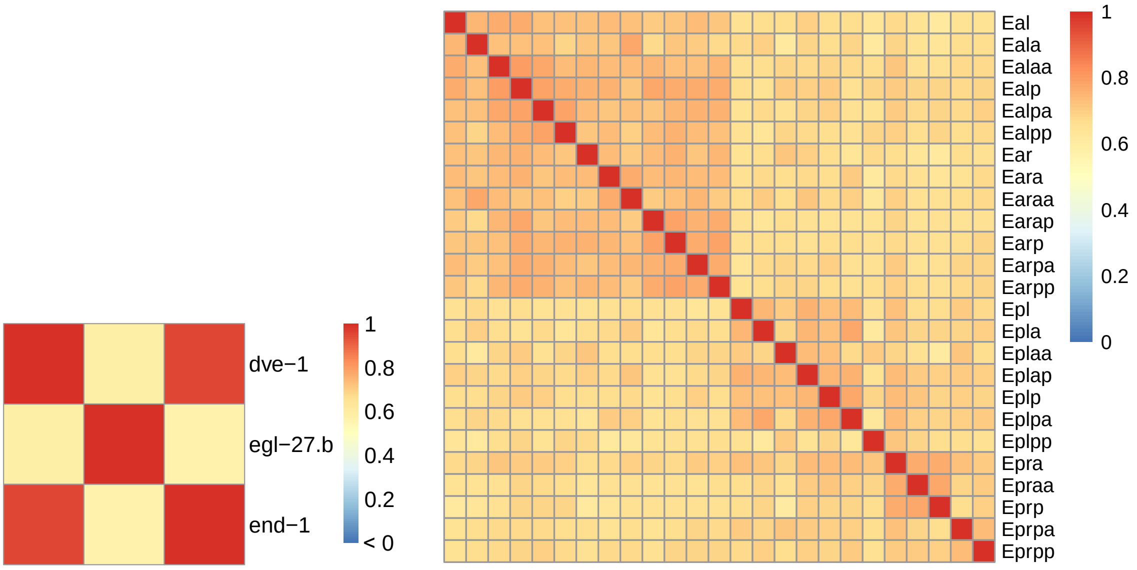

Figure S61: Gene expression heatmap and cell similarity heatmap

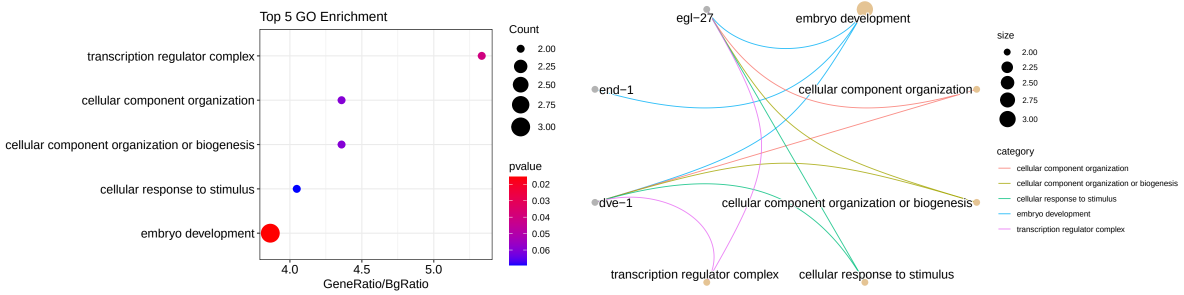

Figure S62: Gene enrichment analysis bubble plot and gene network diagrams for each pathway

•The 8-th biclustering result

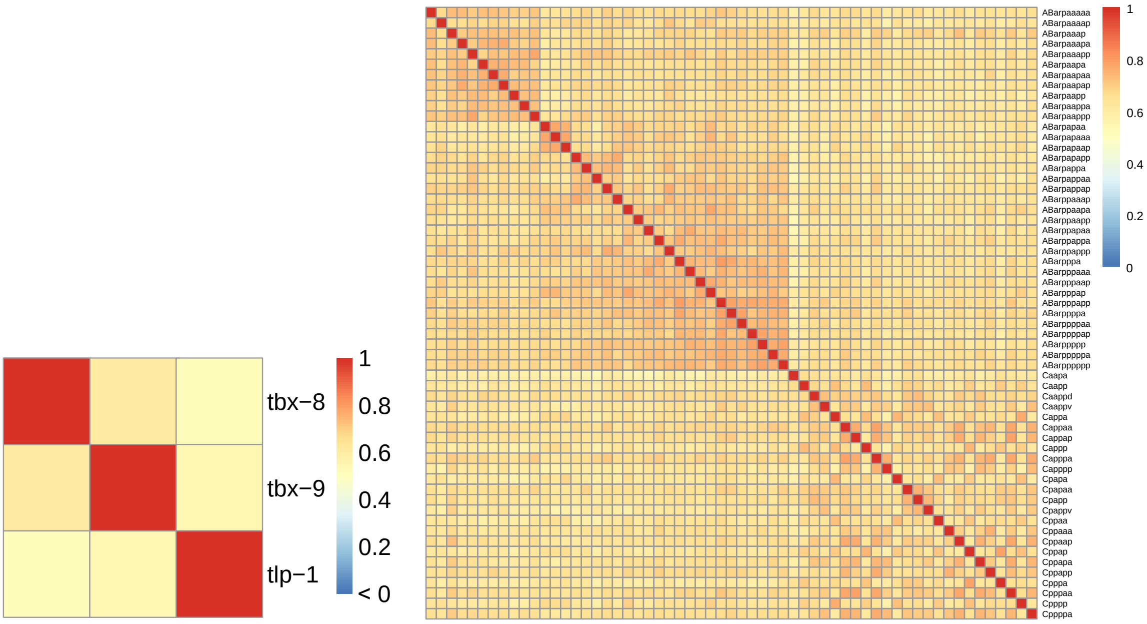

Figure S63: Gene expression heatmap and cell similarity heatmap

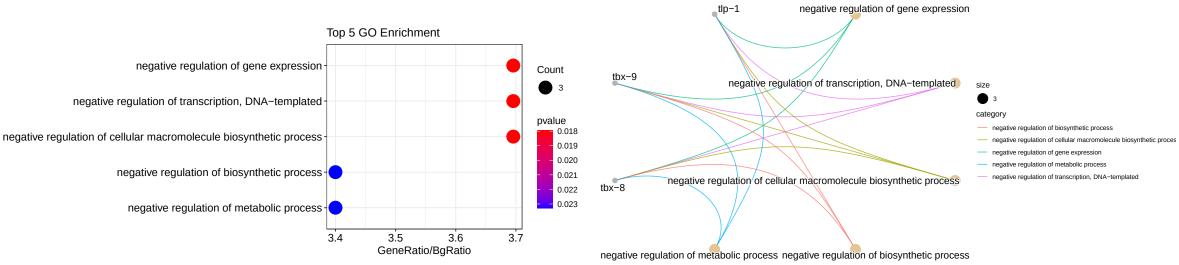

Figure S64: Gene enrichment analysis bubble plot and gene network diagrams for each pathway

•The 9-th biclustering result

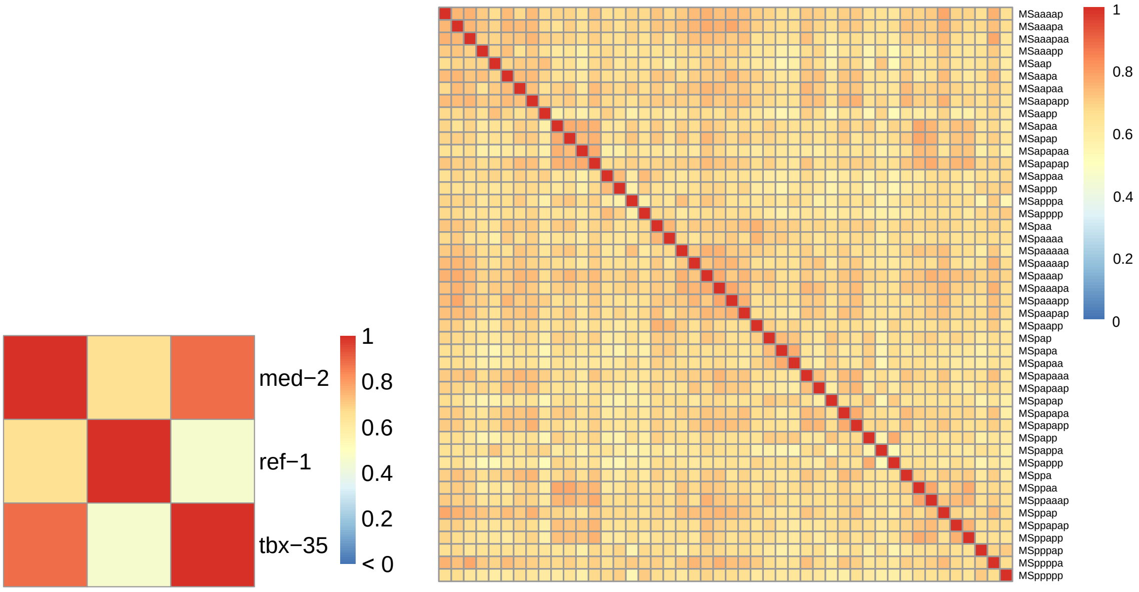

Figure S65: Gene expression heatmap and cell similarity heatmap

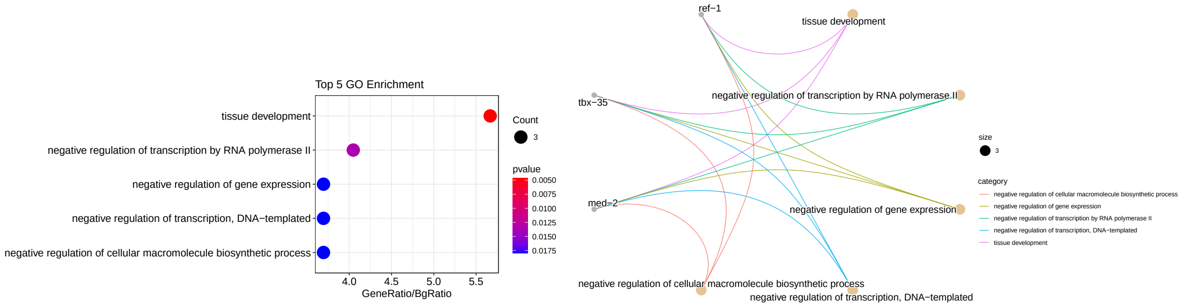

Figure S66: Gene enrichment analysis bubble plot and gene network diagrams for each pathway

•The 10-th biclustering result

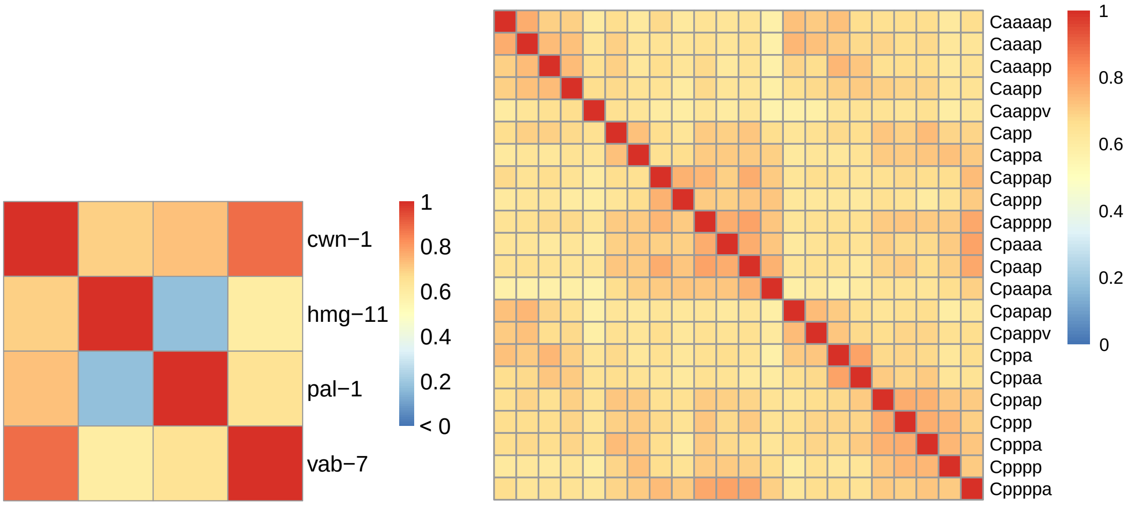

Figure S67: Gene expression heatmap and cell similarity heatmap

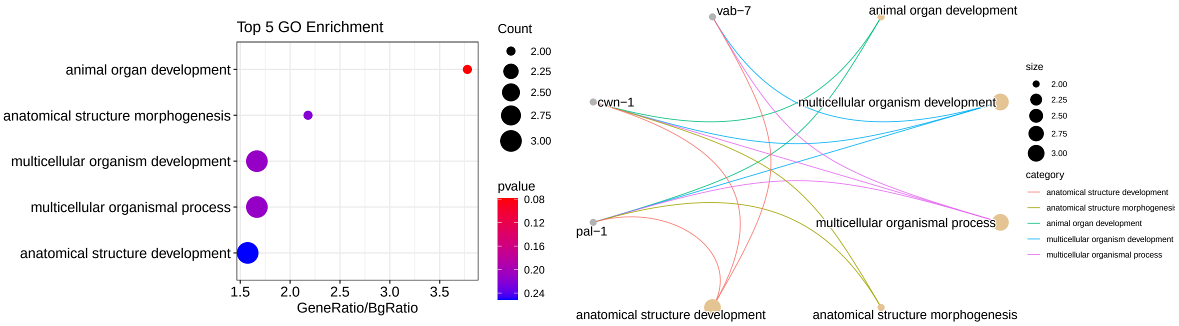

Figure S68: Gene enrichment analysis bubble plot and gene network diagrams for each pathway

References

Cheng, Y. and Church, G. M. (2000). Biclustering of expression data. In *Ismb*, volume 8, pages 93–103.

Hu, J., Zhao, Z., Yalamanchili, H. K., Wang, J., Ye, K., and Fan, X. (2015). Bayesian detection of embryonic gene expression onset in c. elegans. *Annals of Applied Statistics*, 9(2):950–968.

Hubert, L. and Arabie, P. (1985). Comparing partitions. *Journal of Classification*, 2:193–218.

Lazzeroni, L. and Owen, A. (2002). Plaid models for gene expression data. *Statistica Sinica*, pages 61–86.

Liu, X., Yu, T., Zhao, X., Long, C., Han, R., Su, Z., and Li, G. (2023). Arbic: an all-round biclustering algorithm for analyzing gene expression data. *NAR Genomics and Bioinformatics*, 5(1):lqad009.

Murali, T. and Kasif, S. (2002). Extracting conserved gene expression motifs from gene expression data. In *Biocomputing 2003*, volume 8, pages 77–88. World Scientific.

Murray, J. I., Boyle, T. J., Preston, E., Vafeados, D., Mericle, B., Weisdepp, P., Zhao, Z., Bao, Z., Boeck, M., and Waterston, R. H. (2012). Multidimensional regulation of gene expression in the c. elegans embryo. *Genome Research*, 22(7):1282–1294.

Sulston, J. E., Schierenberg, E., White, J. G., and Thomson, J. N. (1983). The embryonic cell lineage of the nematode caenorhabditis elegans. *Developmental Biology*, 100(1):64–119.

Ward Jr, J. H. (1963). Hierarchical grouping to optimize an objective function. *Journal of the American Statistical Association*, 58(301):236–244.

Xie, J., Ma, A., Zhang, Y., Liu, B., Cao, S., Wang, C., Xu, J., Zhang, C., and Ma, Q. (2020). Qubic2: a novel and robust biclustering algorithm for analyses and interpretation of large-scale rna-seq data. *Bioinformatics*, 36(4):1143–1149.
